# AlzDiscovery: A computational tool to identify Alzheimer's disease‐causing missense mutations using protein structure information

**DOI:** 10.1002/pro.5147

**Published:** 2024-09-14

**Authors:** Qisheng Pan, Georgina Becerra Parra, Yoochan Myung, Stephanie Portelli, Thanh Binh Nguyen, David B. Ascher

**Affiliations:** ^1^ The Australian Centre for Ecogenomics, School of Chemistry and Molecular Bioscience University of Queensland Brisbane Australia; ^2^ Computational Biology and Clinical Informatics Baker Heart and Diabetes Institute Melbourne Australia

**Keywords:** AlphaFold2, Alzheimer's disease, machine learning, missense mutation, protein structure, web server

## Abstract

Alzheimer's disease (AD) is one of the most common forms of dementia and neurodegenerative diseases, characterized by the formation of neuritic plaques and neurofibrillary tangles. Many different proteins participate in this complicated pathogenic mechanism, and missense mutations can alter the folding and functions of these proteins, significantly increasing the risk of AD. However, many methods to identify AD‐causing variants did not consider the effect of mutations from the perspective of a protein three‐dimensional environment. Here, we present a machine learning‐based analysis to classify the AD‐causing mutations from their benign counterparts in 21 AD‐related proteins leveraging both sequence‐ and structure‐based features. Using computational tools to estimate the effect of mutations on protein stability, we first observed a bias of the pathogenic mutations with significant destabilizing effects on family AD‐related proteins. Combining this insight, we built a generic predictive model, and improved the performance by tuning the sample weights in the training process. Our final model achieved the performance on area under the receiver operating characteristic curve up to 0.95 in the blind test and 0.70 in an independent clinical validation, outperforming all the state‐of‐the‐art methods. Feature interpretation indicated that the hydrophobic environment and polar interaction contacts were crucial to the decision on pathogenic phenotypes of missense mutations. Finally, we presented a user‐friendly web server, AlzDiscovery, for researchers to browse the predicted phenotypes of all possible missense mutations on these 21 AD‐related proteins. Our study will be a valuable resource for AD screening and the development of personalized treatment.

## 1 INTRODUCTION

Alzheimer's disease (AD), one of the most common forms of dementia and neurodegenerative diseases, has affected over 50 million elderly people in the last century (Knopman et al., 2021). This number is expected to double every 5 years and is projected to exceed 150 million by 2050 (Knopman et al., 2021; Scheltens et al., 2021), bringing a substantial burden on not only the AD‐affected individuals but also their families and society. Two main subcategories were identified based on an age cutoff of 65 years: early‐onset AD (EOAD), accounting for 5%–10% of patients, and late‐onset AD, accounting for 90%–95% of patients (Panegyres & Chen, 2013). AD patients usually first suffer from memory loss, and the symptoms can progressively deteriorate to cognitive disorder, problems with language, and change of personality (Ballard et al., 2011; Dubois et al., 2016; Wattmo et al., 2016). However, there is no cure for this complex disease, despite the expensive treatments to improve the symptoms.

Missense variants are one of the main sources of the dysfunctions of AD‐related proteins. Many rare variants of the amyloid precursor protein (APP), presenilin 1 (PSEN1), and presenilin 2 (PSEN2) increased the ratio of Aβ42/Aβ40 producing higher cellular toxicity (Kelleher 3rd & Shen, 2017). The loss‐of‐function mutations in triggering receptor expressed on myeloid cells‐2 (TREM2) in microglia could also increase the risk of AD up to 4‐fold (Hall‐Roberts et al., 2020; Jin et al., 2014), similar to the risk increment of heterozygous ε4‐apolipoprotein E (APOE) (Gratuze et al., 2018). However, considering the multifactorial pathogenic mechanism of AD, limited resources are available to understand the effects of the mutations of these genes and their phenotypes toward AD. A more comprehensive review was presented in the Supporting Information (Table S1).

Substantial computational methods have been established to estimate the risk of AD on variants since the last decade. The conventional, conservation‐based methods (Adzhubei et al., 2013; Choi & Chan, 2015; Hecht et al., 2015; Ng & Henikoff, 2003) have been used for annotating clinically reported mutations (Zhang et al., 2020), but these methods have poor specificity of diseases. The large deep mutational scanning data (Fowler et al., 2023; Notin et al., 2023) and the deep learning architecture have empowered more sophisticated methods (Brandes et al., 2023; Cheng et al., 2023; Gray et al., 2018; Jagota et al., 2023; Munro & Singh, 2021; Wu et al., 2021) in the recent 5 years, but many of them have limited focus on the impact of variants on protein structural environment. The latest AD‐specific tool, Alz‐Disc (Kulandaisamy et al., 2023), showed promising results in identifying disease‐causing mutations in different AD‐related proteins. This method, however, had no consideration of structural information, and their web server did not provide sufficient interpretation of the annotation of the missense variants.

We have previously shown that using computational tools could help improve the characterization of missense mutations and the prediction of disease phenotypes (Pan et al., 2023). In the current work, we implemented a similar machine learning‐based analysis (Portelli et al., 2021, 2023) on the missense mutations of 21 AD‐related proteins. Qualitative comparisons showed that disease‐causing mutations located in the buried regions tend to have a destabilizing effect on the protein structures. Considering these insights, we initially established a generalized model with comparable performance to current state‐of‐the‐art methods and further improved the performance at the protein‐specific level by tuning the weights in the training process. Our final models also achieved high performance on a clinical validation set, suggesting potential clinical utility. Finally, we developed a user‐friendly web tool to present all the possible missense mutations in these 21 AD‐related proteins. Our work proposed pathogenic molecular drivers toward AD, offering an invaluable resource for a better understanding of the pathology of AD.

## 2 RESULTS

**FIGURE 1:**
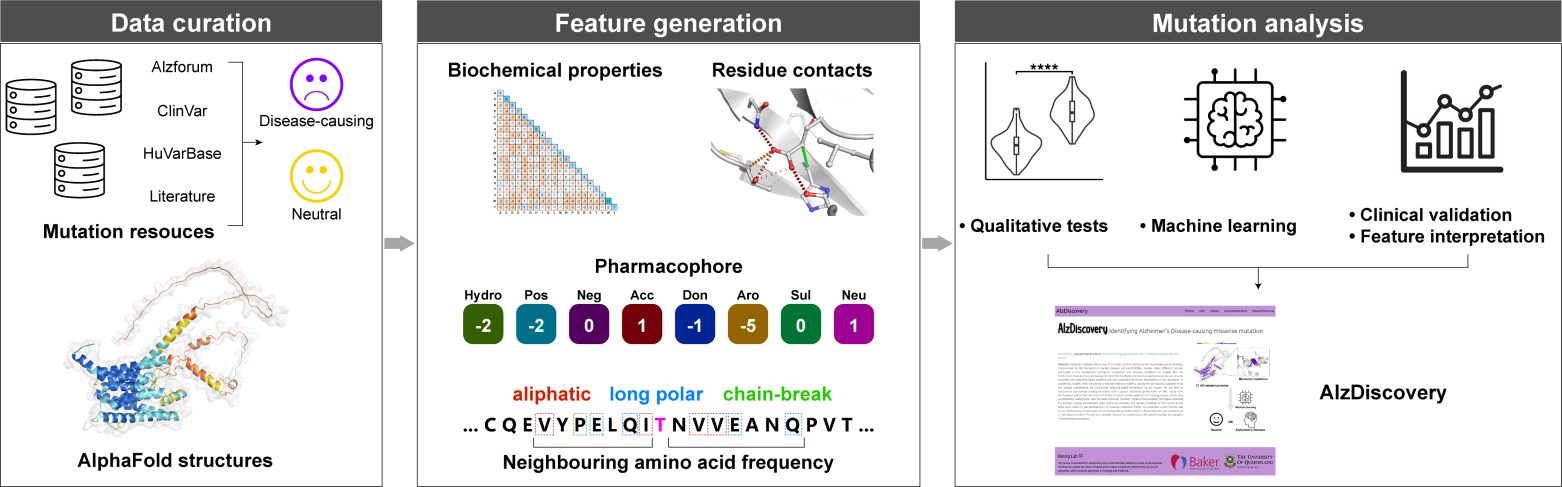
Mutation analysis pipeline to identify Alzheimer's disease (AD)‐causing variants. Missense mutation data were first collected from multiple databases with their labels. Structural information was provided by AlphaFold2 models due to the unavailability of experiment‐determined structures. Then, a wide range of features were computed to describe the effect of mutations. To link these effects with the phenotypes, both qualitative comparisons and the development of machine learning models were implemented, followed by model assessment. Finally, AlzDiscovery, a user‐friendly, interactive web platform, was built for browsing the predicted phenotypes of all the possible missense mutations of each of these proteins.

The mutation analysis pipeline to identify AD‐causing variants was first adopted from previous works (Portelli et al., 2021, 2023) and was further scaled up for 21 AD‐related proteins, whose workflow is depicted in Figure 1. To simplify the name of the protein involved, we used the Gene symbol to refer to the proteins, which could be looked up in the Supporting Information (Table S1). The primary dataset for model training and validation was adapted from previous work with correction of labels of mutations and an independent clinical validation set was curated through ClinVar (Landrum et al., 2014) and literature reviews. We modeled the structures of the target proteins using AlphaFold2 (Jumper et al., 2021) and computed various features to describe the effects of mutations on both sequences and structures using computational biophysical measurements, followed by a qualitative comparison. We then performed a machine learning‐based analysis to accurately characterize these mutations.

### 2.1 Distribution of missense variants of mutation datasets

**FIGURE 2:**
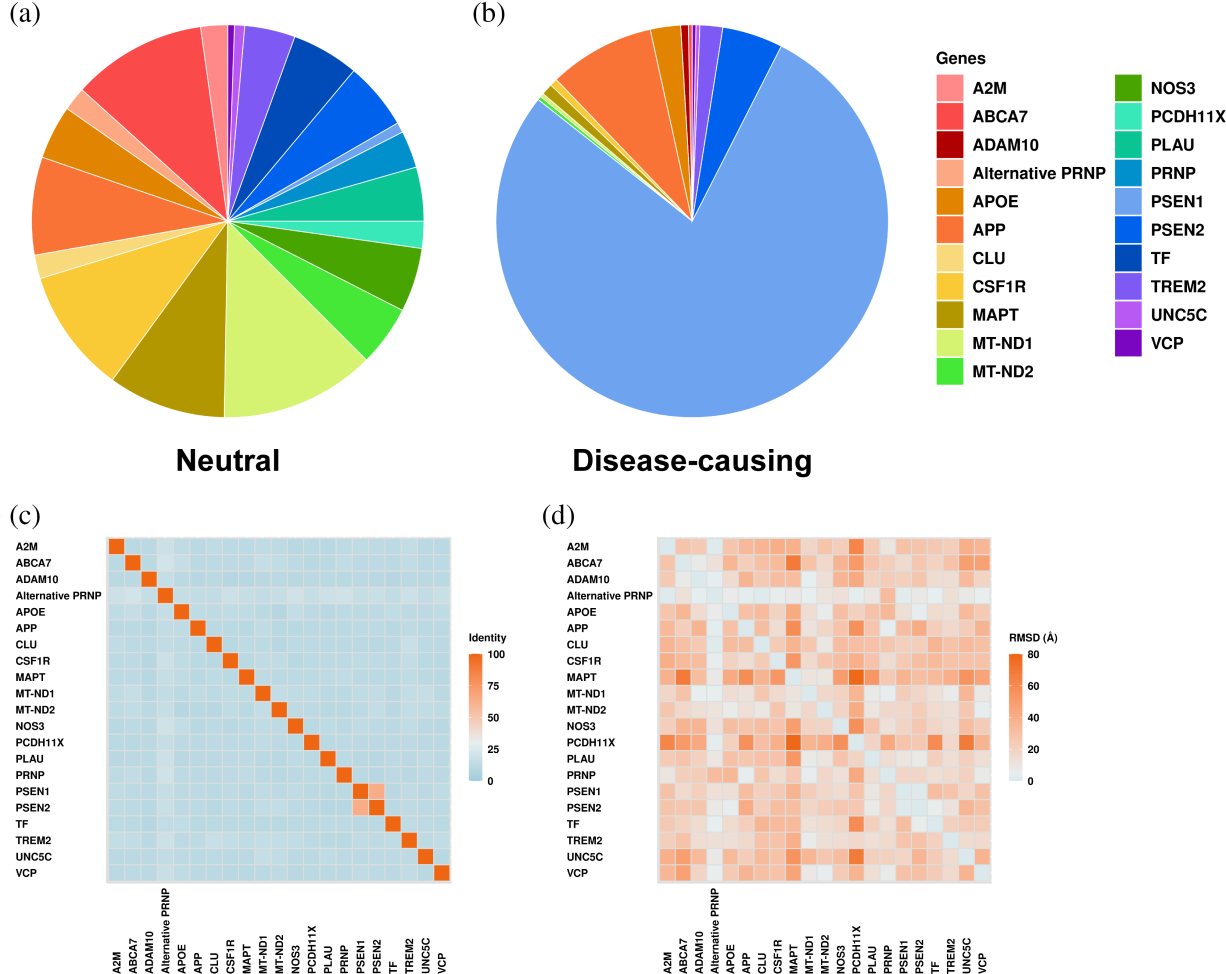
Distribution of the neutral (a) and disease‐causing (b) mutations from 21 Alzheimer's disease (AD)‐related proteins in the primary dataset. Sequence identity (c) and structure similarity measured by all‐atom root mean squared deviation (RMSD) (d) between 21 AD‐related proteins in the dataset are also presented.

In the primary dataset, we curated a balanced dataset (*n* = 680) on 21 AD‐related proteins with a similar distribution of disease‐causing mutations (*n* = 320) and the neutral (non‐AD‐causing) counterparts (*n* = 360) (Table S2, Figure 2). All 21 targets, which are involved in different pathological pathways to AD (Table S1), have different sequences and structures (Figure 2c,d), suggesting a different environment for mutations, except PSEN1 and PSEN2, which share over 60% sequence identity. However, there is a large imbalanced distribution at the protein‐specific levels. Nine out of 21 proteins did not have both phenotypes, eight of which only contained the mutations labeled as neutral. Meanwhile, over 78% of the pathogenic mutations were found in PSEN1 (Figure 2). The other two familial AD (fAD)‐related genes, APP and PSEN2, showed balanced distributions across the different phenotypes.

As for the clinical validation, a dataset with 125 mutations was curated on 11 proteins from the primary dataset, with most mutations labeled as neutral (88%) (Table S3). In addition, this validation set only contained two mutations from PSEN1, whose distribution is completely different from the one in the primary dataset, offering a more challenging clinical validation set for the final model.

### 2.2 Exploring molecular drivers leading to Alzheimer's disease

**FIGURE 3:**
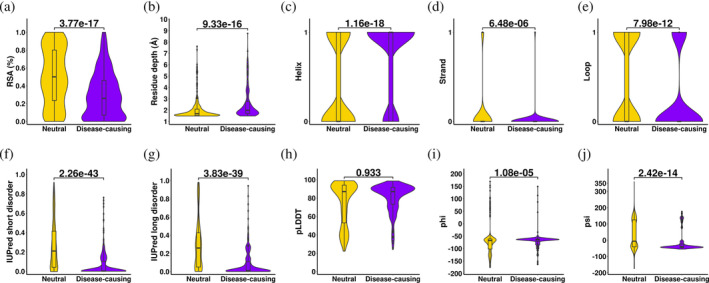
Qualitative comparisons on the protein structural environment to identify molecular drivers leading to AD. The two‐tailed Wilcoxon Sign‐rank tests were performed to compare the structural properties at the mutation site, including relative solvent accessibility (RSA) (a), residue depth (b), secondary structure type (c–e), protein disorderness (f–g), residue confidence score from AlphaFold2 (h), and torsion angle of peptide bonds (i, j), at 5% significance level.

When comparing the biophysical measurements between disease‐causing and neutral mutations, a qualitative comparison was first performed on the protein structural environment, to investigate the potential impact of mutations. We noticed that the pathogenic mutations are predominantly located in the protein core, while neutral mutations are more solvent exposed (RSA: *p*‐value = 3.77 × 10^−17^, residue depth: *p*‐value = 9.33 × 10^−16^, Figure 3a,b). In addition, there are differences in secondary structure at the mutation site (Figure 3), particularly on the helices (*p*‐value = 1.16 × 10^−18^), as around 50% of target proteins are transmembrane proteins. Disease‐causing mutations are enriched in the structured regions, while the neutral ones are in the disordered regions (IUPred short score: *p*‐value = 2.26 × 10^−43^, IUPred long score: *p*‐value = 3.83 × 10^−39^, Figure 3f,g). Combining these differences in the structural environment at the mutation site, we suspect the pathogenic mutations tend to be located in the buried, helical regions of the protein structure, and they may alter the hydrophobic environment, which could cause a drastic effect on protein folding, such as the change of protein stability.

**FIGURE 4:**
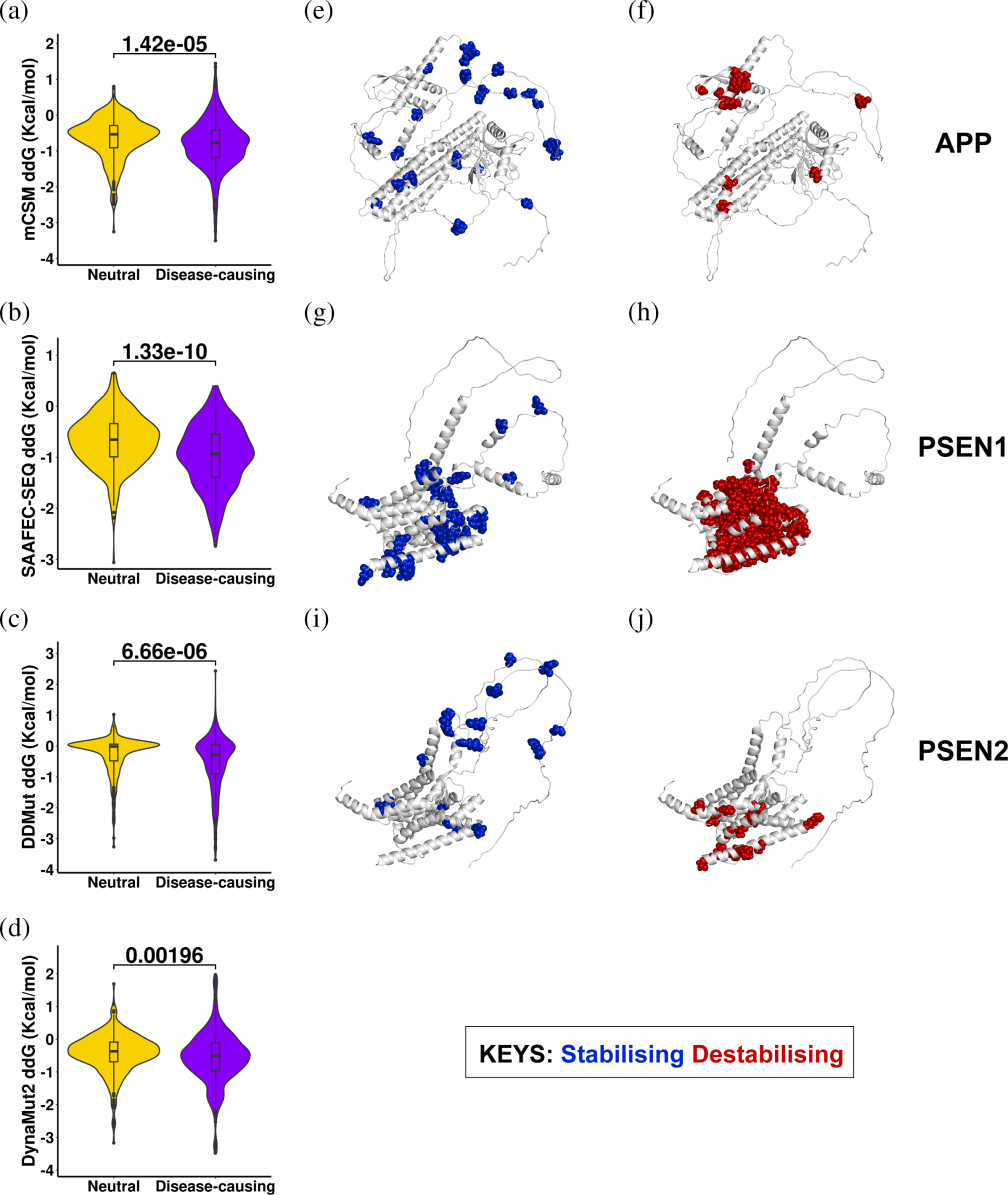
Pathogenic molecular drivers leading to AD. Qualitative analyses were performed to compare the effect of mutations on the change of protein stability computed by mCSM‐Stability (a), SAAFEC‐SEQ (b), DDMut (c), and DynaMut2 (d), measured by ΔΔG (Kcal/mol) with zero as a cutoff (ΔΔG > 0: stabilizing; ΔΔG < 0: destabilizing). Qualitative analysis was implemented using the two‐tailed Wilcoxon Sign‐rank test at a 5% significance level. Detailed structural analysis was implemented to visualize the mutations with stabilizing/destabilizing effects on three familial AD‐related genes, APP (e and f), PSEN1 (g and h), and PSEN2 (i and j).

We therefore compared and noticed that there is a significant difference in the change of protein stability between neutral and disease‐causing mutations (*p*‐value <0.002, Figure 4a–d), consistent across four predictors, namely mCSM‐Stability (Pires et al., 2014), SAAFEC‐SEQ (Li et al., 2021), DDMut (Zhou et al., 2023), and DynaMut2 (Rodrigues et al., 2021). The AD‐causing group displays a stronger destabilizing effect, containing more mutations with negative ΔΔG values. Further to that, we structurally analyzed the mutations in those three fAD‐related proteins (APP, PSEN1, and PSEN2) as a case study, and confirmed that a majority of mutations cause destabilizing effects (APP: 49.12%, PSEN1: 73.12%, and PSEN2: 52.78%), suggesting the change of stability upon mutations as a pathogenic molecular driver (Figure 4e–j), consistent with previous experimental findings (Grant et al., 2007; Rezaei‐Ghaleh et al., 2023; Roher et al., 1993). Our results provide a hypothesis that the missense mutations in APP monomers carrying destabilizing effects may introduce more protein aggregation of Aβ peptides to maintain protein stability.

**FIGURE 5:**
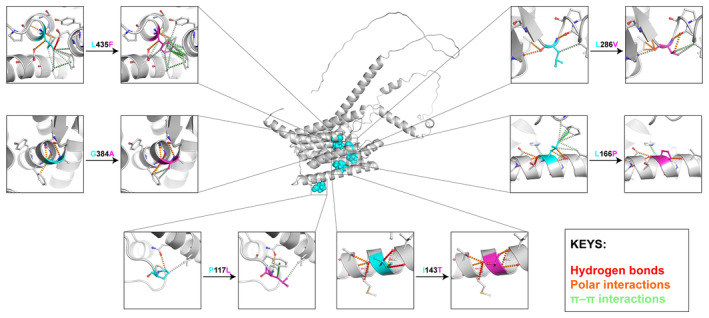
Analysis of the intramolecular residue interaction of six representative mutations of PSEN1 protein, namely P117L, I143T, L166P, L286V, G384A, and L435F. Wild‐type residues were colored as cyan, while the mutant residues were colored as magenta. The hydrogen bonds were represented by red dashed lines. The polar interactions were represented by orange dashed lines. The π–π interactions were represented by green dashed lines.

To further explore the intramolecular changes upon mutations, six representative mutations from the PSEN1 protein, which is well identified to be AD‐causing in the previous study (Do et al., 2023), were selected for further investigation (Figure 5). In general, these mutant structures present considerable differences in the structural environment. The change of biochemical properties caused by mutations introduced the loss of π–π interactions, especially in P117L, L166P, and L435F. The P177L and L286V were located in the flexible loop regions, which cause less impact on protein stability based on the prediction of DDMut (ΔΔG_P117L_ = 0.05 Kcal/mol and ΔΔG_L286V_ = 0.01 Kcal/mol, respectively), while the other four mutations (ΔΔG_I143T_ = −0.07 Kcal/mol, ΔΔG_L166P_ = −0.08 Kcal/mol, ΔΔG_G384A_ = −0.18 Kcal/mol, and ΔΔG_L435F_ = −2.11 Kcal/mol) could potentially disrupt protein folding, negatively affect the conformational changes to active state of γ‐secretase, and further reduce the activity of cleavage of APP, consistent with previous findings (Do et al., 2023; Somavarapu & Kepp, 2016).

### 2.3 Predictive performance to identify disease‐causing mutations using machine learning

Given the bias on the destabilizing effect of disease‐causing mutations, we tried to build a machine‐learning model to better characterize the pathogenic variants leading to AD. The primary dataset was first divided into training (60%), validation (20%), and blind test (20%) sets, ensuring non‐redundancy between datasets according to unique protein position. Seven machine learning algorithms were first employed to elucidate the association between different molecular features and phenotypes of mutations, and the algorithm with the best predictive performance was selected and further optimized using greedy feature selection (details in Section 4). Our initial generic model based on the Gradient Boosting algorithm achieved high performance on both 10‐fold cross‐validation (10‐CV) (area under the curve (AUC) = 0.94, Matthew's correlation coefficient (MCC) = 0.79) and the non‐redundant blind test (AUC = 0.94, MCC = 0.75) (Table S4). After configuring the sample weights in the training process to improve the attention for target proteins, our enhanced model obtained a higher overall performance on the blind test (AUC = 0.95, MCC = 0.79, Table 1). The consistent performance of the blind test on sensitivity (0.88), specificity (0.92), and precision (0.90) suggests our model was not biased toward a specific phenotype.

**TABLE 1:** Performance of AlzDiscovery of different validation.

Test type	BACC	F1‐score	MCC	Sensitivity^a^ (recall)	Precision^a^	Specificity^a^	AUC
10‐CV	0.89	0.88	0.79	0.86	0.91	0.92	0.95
Blind test	0.90	0.89	0.79	0.88	0.90	0.92	0.95
Clinical validation	0.68	0.41	0.32	0.47	0.37	0.89	0.70

^a^ In this work, the label “Disease‐causing” was considered as positive, while “Neutral” was considered as negative.

In addition to a small improvement in the generalized performance, the accuracy at the protein‐specific level for the target protein was significantly improved (Figure S1), especially on those proteins with small sample size of mutations in the primary dataset (alternative PRNP and NOS3). The performance on APOE has a small drop after weight tuning, while the performance on the other proteins remains the same. Our model fails to predict the pathogenic mutations in ADAM10, which is likely due to the extremely small number of samples (*n* = 2) in the dataset.

To assess the generalizability of our model at the protein‐specific level, a leave‐one‐protein‐out validation was conducted (Table S5). We noticed that there is a large overall performance deterioration, with AUC down to 0.65. This decrease in performance can be attributed to the imbalanced distribution of data within our primary dataset, as over 78% of all mutations were present in one protein, PSEN1. In excluding this protein during leave‐one‐protein‐out validation, our previous accuracy of 0.97 dropped to 0.21, showing the significant contribution of these mutations to the overall performance. Apart from this, in considering 21 different genes, different mechanisms contributing to AD have been assessed in our initial model. During leave‐one‐protein‐out, removing specific proteins could underrepresent minor mechanisms of AD, which would also be reflected in lower accuracy.

We then applied the enhanced model to the clinical validation set to assess model generalizability and the clinical utility, excluding APOE, for which we applied the generic model due to the performance drop obtained through the enhanced model. Despite an overall performance deterioration, our model still achieved a relatively reasonable performance (AUC = 0.70 and MCC = 0.32) (Table 3), indicating a certain degree of generalizability of the model. The model still achieved a relatively balanced capability on classifying two different phenotypes (sensitivity = 0.47 and precision = 0.37), but the high specificity (0.92) suggests a certain bias in the predictions toward Neutral phenotypes. At the protein‐specific level, the performance of some proteins, such as CSF1R, PRNP, and VCP, was improved by around 4%–22% (Figure S1D). However, there was a performance drop when predicting the mutations from PSEN2.

### 2.4 Performance comparison with the state‐of‐the‐art methods

**FIGURE 6:**
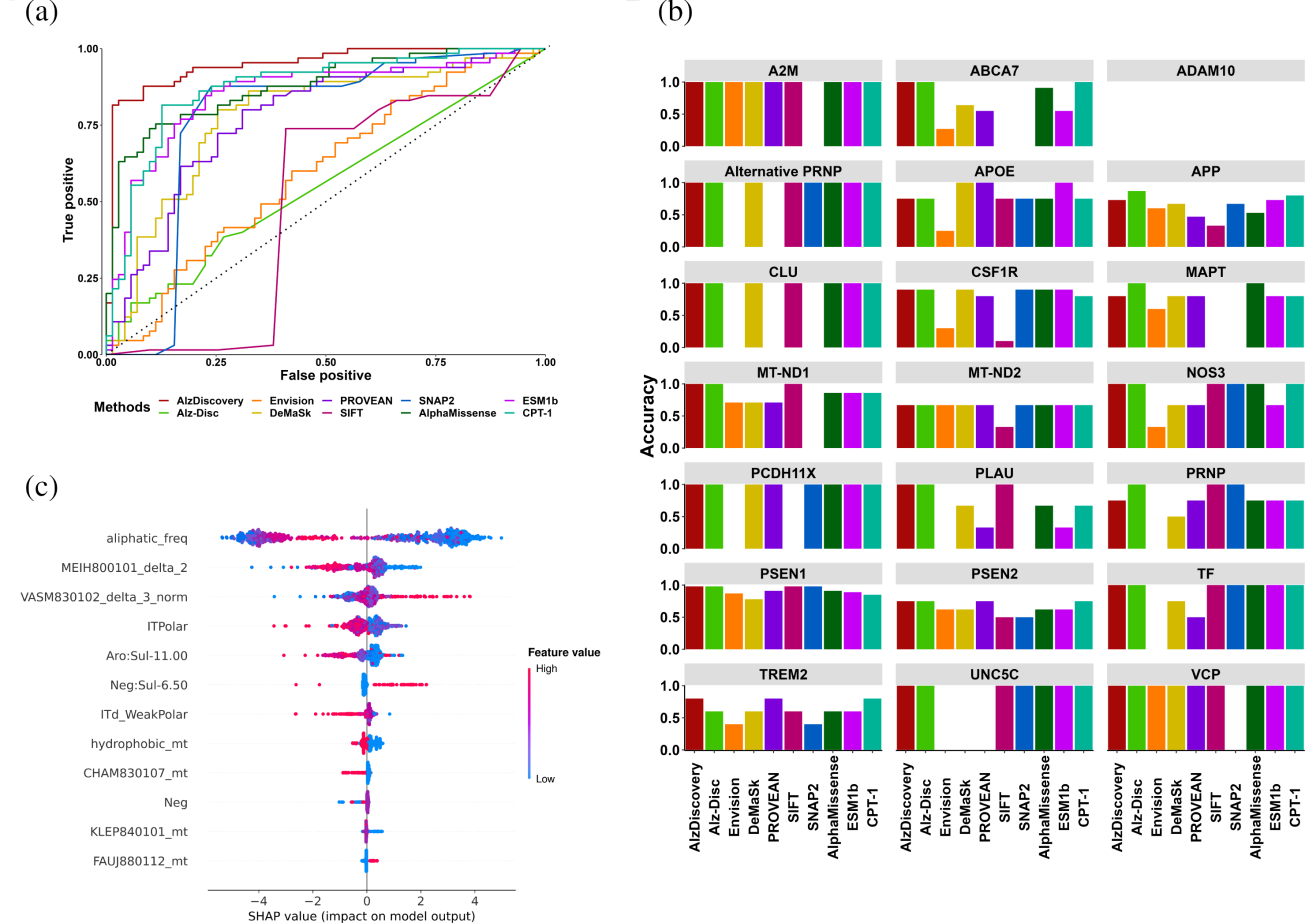
Predictive performance comparisons and model interpretation. Predictive performance of AlzDiscovery and the other variant effect predictors on the blind test is shown in the receiver operating characteristic (ROC) curve (a), and a performance at the protein‐specific levels is compared using accuracy due to the imbalance of mutation phenotypes of each protein (b). Feature importance of the AlzDiscovery model was interpreted using SHapley Additive exPlanations (SHAP) method (c). Features were ordered from top to bottom based on their contributions to the models. To interpret the plot, the contribution of features toward different labels is represented by the direction of the *x*‐axis (negative: disease‐causing; positive: neutral), while the numeric value of the feature is represented by the color of each data point (red: High; blue: Low). Combining these two aspects, the contributions of each feature could be interpreted.

When comparing our method with the current advanced variant effect predictors, we found that our tool achieved comparable performance on the blind test with the AD‐specific approach, Alz‐Disc, across different metrics (Table 2, Figure 6). Though the comparison of performance of the blind test between our method and Alz‐Disc may not be fair due to the redundancy caused by different divisions of training and test sets, our model achieved a much higher AUC (AUC = 0.95) than that of Alz‐Disc (AUC = 0.55), suggesting a higher confidence of the predicted phenotypes from our model. At the protein‐specific level, while the Alz‐Disc achieved a better accuracy on three proteins, APP, MAPT, and PRNP, our method is capable of accurately identifying the mutations from TREM2, a newly proposed AD biomarker, as we corrected the mutation labels of TREM2 in the primary dataset (Figure 5B). In terms of the independent validation, our model presented a much better performance (AUC = 0.70 and MCC = 0.32) in the clinical benchmark, compared with Alz‐Disc (AUC = 0.54 and MCC = 0.18) (Table 3). Particularly, our model could better classify mutations in well‐known biomarkers, such as APP, APOE, MAPT, and TREM2 (Table S6). As we trained our model by integrating structure‐based features, this may suggest the importance of considering the effects of mutation in a three‐dimensional environment to enhance the robustness.

**TABLE 2:** Performance comparison on the blind test of the primary dataset.

Method	Type	BACC	F1‐score	MCC	Sensitivity (recall)	Specificity	Precision	AUC
AlzDiscovery	AD‐specific	0.90	0.89	0.79	0.88	0.92	0.90	0.95
Alz‐Disc	AD‐specific	0.92	0.91	0.84	0.88	0.96	0.95	0.55
DeMaSk	Deep mutation scanning‐based	0.73	0.71	0.47	0.69	0.77	0.74	0.78
Envision	Deep mutation scanning‐based	0.57	0.64	0.16	0.83	0.31	0.52	0.60
PROVEAN	Conservation‐based	0.73	0.74	0.46	0.80	0.66	0.68	0.77
SIFT	Conservation‐based	0.66	0.67	0.32	0.72	0.59	0.62	0.51
SNAP2	Conservation‐based	0.67	0.71	0.37	0.88	0.46	0.60	0.77
AlphaMissense	Deep learning‐based	0.82	0.80	0.65	0.75	0.89	0.86	0.88
ESM1b	Deep learning‐based	0.80	0.79	0.59	0.82	0.77	0.77	0.85
CPT‐1	Deep learning‐based	0.84	0.83	0.68	0.80	0.87	0.85	0.87

**TABLE 3:** Performance comparison on the clinical validation.

Method	Type	BACC	F1‐score	MCC	Sensitivity (recall)	Precision	Specificity	AUC
AlzDiscovery	AD‐specific	0.68	0.41	0.32	0.47	0.37	0.89	0.70
Alz‐Disc	AD‐specific	0.61	0.30	0.18	0.40	0.24	0.83	0.54
DeMaSk	Deep mutation scanning‐based	0.42	0.16	−0.11	0.53	0.10	0.31	0.64
Envision	Deep mutation scanning‐based	0.48	0.20	−0.05	0.87	0.12	0.09	0.59
PROVEAN	Conservation‐based	0.54	0.23	0.07	0.93	0.13	0.15	0.57
SIFT	Conservation‐based	0.41	0.10	−0.12	0.2	0.07	0.62	0.61
SNAP2	Conservation‐based	0.52	0.21	0.03	0.60	0.13	0.45	0.54
AlphaMissense	Deep learning‐based	0.39	0.14	−0.15	0.47	0.09	0.32	0.40
ESM1b	Deep learning‐based	0.53	0.22	0.04	0.73	0.13	0.33	0.50
CPT‐1	Deep learning‐based	0.61	0.27	0.14	0.73	0.16	0.48	0.58

Our enhanced model outperformed the conservation‐based methods (SIFT (Ng & Henikoff, 2003), SNAP2 (Hecht et al., 2015), and PROVEAN (Choi & Chan, 2015)) on the blind test (Table 2, Figure 6). Meanwhile, our approach also presents a better performance on both the blind test and independent validation set than the ones of deep mutation scanning (DMS)‐based methods and deep learning‐based methods, including the latest approach AlphaMissense (AUC_blind‐test_ = 0.88 and AUC_validation_ = 0.40) (Cheng et al., 2023). These generalized variant effect predictors were established to evaluate the fitness of the residue substitution, and therefore may not have enough specificity on neurodegenerative diseases such as AD. However, it should be emphasized that those new deep‐learning‐based methods, including AlphaMissense, ESM1b (Brandes et al., 2023), and CPT‐1 (Jagota et al., 2023), present a higher generalizability to identify AD‐causing mutations, which could be due to the use of feature vectors cooperated with protein structural information.

### 2.5 Model interpretation

Since both our generic and protein‐specific models obtained good performance, we then tried to explore the contributions of each feature to gain a better understanding of the instrumental factors to identify AD‐causing mutations (Figure 6c). Our models leverage 12 features describing the mutation from different aspects. The feature with the highest contributions is the neighboring aliphatic amino acid frequency (*aliphatic_freq*), consistent with one of the features of Alz‐Disc (Kulandaisamy et al., 2023). Combining this with another three features, namely the structural polar contact at theWT mutation site (*ITPolar*), the difference of structural weak polar contact (*ITd_WeakPolar*), and the hydrophobic mutant (*hydrophobic_mt*), we suspect that the increment of hydrophobicity at the mutation site may not only impact protein stability but promote protein aggregation, consistent with previous studies (Dasuri et al., 2010; Kim & Hecht, 2006). There are five other features derived from AAindex (*MEIH800101_delta_2*, *VASM830102_delta_3_norm*, *CHAM830107_mt*, *KLEP840101_mt*, and *FAUJ880112_mt*), which account for different biochemical properties of 20 natural amino acids, namely the change of hydrophobic behavior in two neighboring amino acids (Meirovitch et al., 1980), the change of computed conformational state in neighboring region (Vasquez et al., 1983), and three properties of mutant amino acids (the intermolecular force and steric parameters (Charton & Charton, 1983), the net charge (Klein et al., 1984), and the negative charged (Klein et al., 1984), respectively). Two structural features from graph‐based signatures (*Aro:Sul‐11.00* and *Neg:Sul‐6.50*) explain the structural environment of atoms with aromatic, sulfur, and negative pharmacophores, while the feature *Neg* represents the difference of negative pharmacophores between WT and mutant amino acids.

In brief, these features mainly inform the importance of the hydrophobic environment at the mutation site, the biochemical properties in the neighboring region, and the change of polar residue contacts. The change of hydrophobicity at the mutation environment could be particularly crucial to the aggregation of Aβ and the formation of amyloids, agreeing with our qualitative analysis and other previous works (Sgourakis et al., 2007; van Gils et al., 2020).

### 2.6 Saturation mutagenesis and web server

**FIGURE 7:**
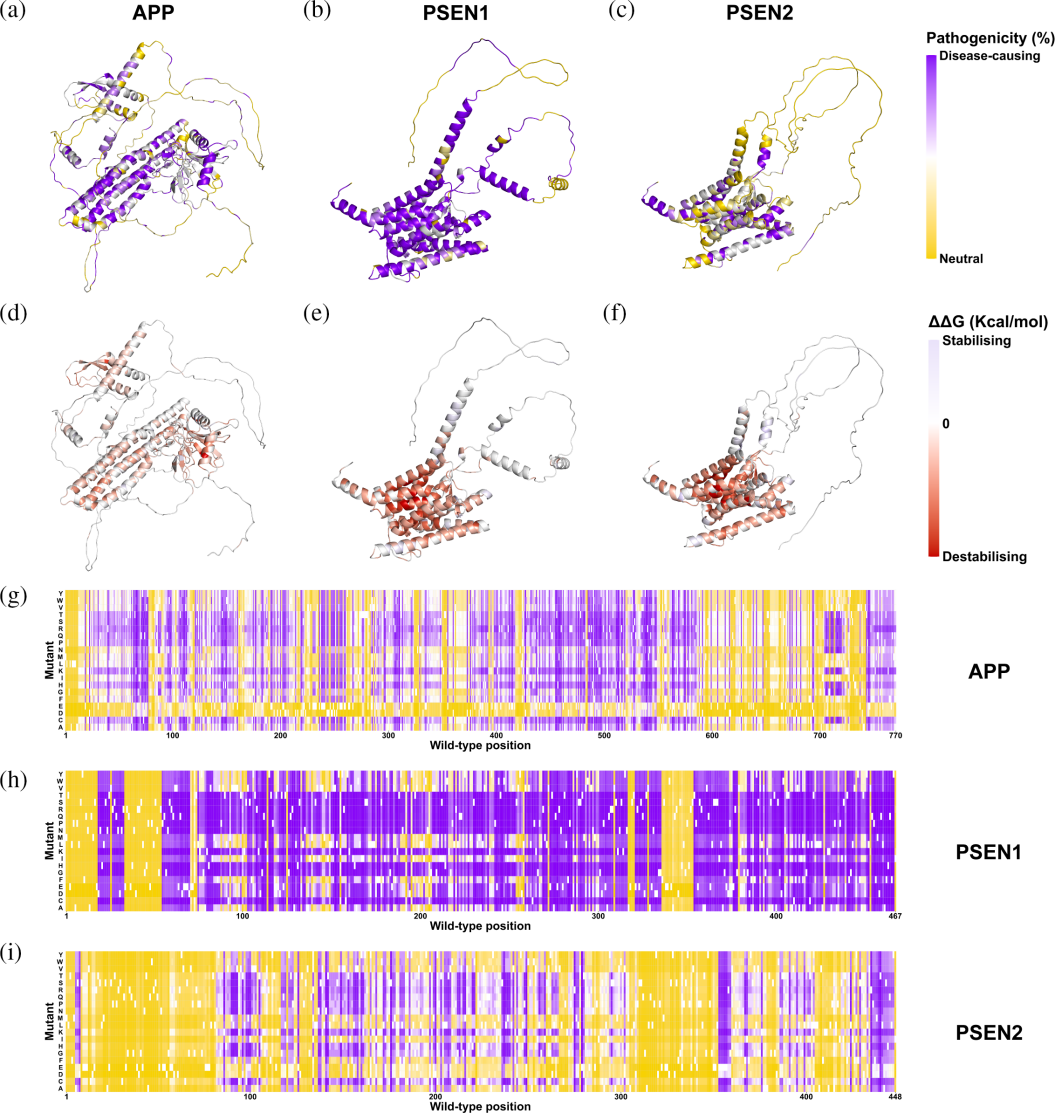
Mutation landscape leading to AD of three familial AD‐related proteins, APP, PSEN1, and PSEN2. Protein structures are colored based on two color schemes, namely the average probability leading to diseases of all possible missense mutations at each mutation site (a–c) and the change of protein stability upon mutations at the mutation site (ΔΔG, d–f), while the heatmaps present detailed results of different mutants at each site of three proteins (g–i).

Finally, we applied our final models with the best performance at the protein‐specific level to generate the predictions of all possible missense mutations for these 21 AD‐related proteins. To be specific, we applied the enhanced protein‐specific models to all proteins for *in silico* saturation mutagenesis, excluding APOE and PSEN2 because of the performance deterioration of their weighted model, on which we applied the initial generic model. Here we present the mutation landscape of three fAD‐related proteins, APP, PSEN1, and PSEN2 (Figure 7), and three strong risk factors, APOE, MAPT, and TREM2 (Figure S2), while the rest are available on the AlzDiscovery web server https://biosig.lab.uq.edu.au/alzdiscovery/.

We initially noticed that the pathogenic variants are enriched in the region where mutations tend to destabilize protein folding (Figures 7a–f and S2A–F), consistent with the qualitative analyses of computational biophysical measurements. Following detailed analysis of the intramolecular residue contacts in the fAD‐related genes (Figure S3) suggests that the destabilizing effect is due to the change in interaction profiles, such as the alteration of hydrophobic and polar contacts. Our structure‐based model, however, may not be capable of a thorough explanation of all mutations (Figure S3A) due to the complexity of AD. A similar analysis on the change of residue contacts is found through the web server.

The pathogenic area tends to form clusters at some specific mutation sites, where most residue substitutions could cause pathogenicity. This is mainly because a number of features in the models are capturing the residue environment at the mutation loci, rather than a more detailed change between WT and mutant amino acid. These selections of features are associated with the bias of mutation distribution in the dataset, as there are not enough mutations at the same mutation site of a protein with different phenotypes, resulting in the model trying to identify the disease‐causing regions instead of the disease‐causing mutations. In addition, there is a bias to neutral phenotypes of those mutations toward the negative charge amino acids, particularly obvious in APP, PSEN2, and TREM2 (Figures 7g,j and S2F), due to the use of feature *FAUJ880112* (Figure 6c). The limited mutation types may also lead to a neutral bias on negative charge mutants. In terms of APOE and TREM2, disease‐causing mutations are mainly clustered at the helical regions (Figure S2), consistent with our results on qualitative analysis. A different pattern was noticed in the tau pathology. The mutation landscape on the MAPT shows only a relatively small number of pathogenic mutations clustered at mutation site P587 (Figure S2B). This indicates that missense mutations on tau protein may not be the main drivers of AD but other dementia, such as frontotemporal dementia with Parkinsonism linked to chromosome 17 (FTDP‐17), consistent with experimental studies (Alonso Adel et al., 2004; Bhaskar et al., 2005).

## 3 DISCUSSION

The complicated pathogenic mechanism of AD has been significantly studied over the last century, which manifests different biomarkers, including the accumulation of Aβ peptides, tau pathology, dysregulation of lipidology, and the activation of innate immune reaction in the human central nervous system. Missense mutations on these AD‐related proteins have been reported as one of the key pathogenic drivers (Bossaerts et al., 2022; Brownjohn et al., 2018; Sherrington et al., 1996; Wakutani et al., 2004). In the current work, we have characterized the effects of mutations on 21 AD‐related proteins with a structural insight using computational biophysical methods and machine learning. Our model achieved a relatively robust performance on different benchmarks, and the interpretation of the models revealed that missense mutations could alter the hydrophobic environment, protein stability, and polar residue interactions on AD‐related proteins, which could be important molecular drivers leading to disease. A user‐friendly and interactive web platform was established for all researchers to browse the predicted phenotypes and visualize the changes in the protein structures.

We propose two important mutational risk factors, namely the hydrophobic environment at the mutation site and the changes in protein stability caused by mutation. These two factors are both associated with the protein intact folding, which is crucial for protein functions. The authors of Alz‐Disc also proposed a similar idea (Kulandaisamy et al., 2023) by interpreting the neighboring amino acid frequency, but we directly used the biophysical measurements on the protein structures and further supported this hypothesis. In addition to the effect on a monomeric structure, the change in hydrophobicity and protein stability are also key factors in protein aggregation. We could have implemented more biophysical measurements, such as the protein–protein interaction of Aβ peptides, and the lipid binding of APOE (Frieden et al., 2017) and TREM2 (Wang et al., 2015). These in‐depth analyses, however, were hindered by the complexity and scale of this work.

By improving the implementation of the mutation analysis pipeline in the previous work (Portelli et al., 2021; Portelli et al., 2023), we managed to investigate the effect of missense mutations and predict the disease‐related phenotypes on multiple proteins, by tuning the sample weights of the mutation from a target protein. This method could further improve the performance at the protein‐specific level, but we also noticed the performance deterioration on two proteins, APOE and PSEN2, due to the bias of features, such as the biased representation of negative‐charged mutant amino acids. We therefore use the generic models to perform saturation mutagenesis for these two proteins. This is because while we believe the weight‐tuning approach could improve the predictive performance (Fang et al., 2022), we also aimed to give the best predictions on these mutations for clinical use.

Further to this, there were three limitations in this work that were faced due to the lack of variant data availability. Firstly, the imbalanced distribution of mutations across proteins studied in the primary dataset resulted in a bias of our model, which is exemplified by the performance deteriorations on the leave‐one‐protein‐out test (Table S5). Similarly, the insufficient coverage of different types of residue substitutions led to a relative deficiency in performance, especially the performance in predicting the mutations toward negative‐charged amino acids (Figure 7g–i). Meanwhile, there is no data to describe the interactions among the mutations and AD‐related genes, which means that our model did not capture the complex gene regulation network associated with the pathogenesis of AD. Therefore, the mutation analysis toward AD can be improved when more high‐quality data becomes available in the future.

Understanding the potential risk of AD could be useful for patient monitoring in the early stages, especially for EOAD and fAD. Clinical researchers could easily access our web tool and retrieve the results from computational saturation mutagenesis. Further to that, an interactive structure viewer is available to present the residue interactions, the regions with decreased protein stability, and other biochemical properties. Thus, we believe that our work could be an invaluable resource for not only further AD research but also clinical application. Personalizing treatment could be designed by leveraging the mutation information from our saturation mutagenesis, as a pioneering study has shown the feasibility of using small‐molecule drugs to stabilize Aβ (Lohr et al., 2022).

## 4 MATERIALS AND METHODOLOGY

### 4.1 Data curation and protein structural information

#### 4.1.1 Primary dataset

We leveraged the dataset curated by previous work (Kulandaisamy et al., 2023) and implemented data cleaning. According to the previous work, mutations associated with AD from multiple resources, including Alzforum (Kinoshita & Clark, 2007), Humsavar from UniProt (Apweiler et al., 2004), ClinVar (Landrum et al., 2018), dbSNP (Smigielski et al., 2000), and HuVarBase (Ganesan et al., 2019), were extracted and labeled as disease‐causing and neutral respectively according to the annotations from the databases. We next performed a data cleaning on this original dataset, which involved the removal of synonymous mutations, and missense mutations that did not map to UniProt canonical sequences. All mutations located in the fAD‐related genes (APP, PSEN1, and PSEN2) and AD risk factors (APOE, MAPT, and TREM2) were further manually checked to ascertain their reliability. Notably, all TREM2 mutations were labeled as Neutral in the original dataset, so we carefully corrected the mutation phenotypes by checking the contradictory labels in these resources. In the final dataset, six TREM2 mutations (R47H, R62H, T66M, R136Q, H157Y, and T223I) were marked as Disease‐causing, as reported in the literature (Jin et al., 2014; Sayed et al., 2021; Sirkis et al., 2016). After data cleaning, the final dataset consisted of 680 mutations on 21 AD‐related proteins (available in the Supplementary Files), which was used for model development and optimization.

#### 4.1.2 Clinical validation

To assess the clinical utility of our model, missense mutations of these 21 AD‐related proteins were collected from ClinVar via precise phenotyping based on clinical conditions from literature or the American College of Medical Genetics and Genomics (ACMG) guidelines (Richards et al., 2015). Redundant mutations between the primary dataset and clinical validation set were removed. The final clinical validation set contains 125 mutations on 11 proteins, which is available in the Supplementary Files.

#### 4.1.3 Structure curation

Protein structural models were generated to improve the performance of identifying pathogenic mutations toward AD. Due to the unavailability of the experiment‐determined structure of most proteins of interest, protein structures were generated by the latest AlphaFold2 (Jumper et al., 2021) using the canonical protein sequences with template date *2022‐03‐02*. Models with the highest confidence score (predicted Local Distance Difference Test, pLDDT (Mariani et al., 2013)) were selected for feature generations.

### 4.2 Feature generations

Numerous sequence‐ and structure‐based features were computed to annotate the effects of mutations in various aspects, including biochemical properties, protein biophysical properties, local residue environment, and functionality assessment.

#### 4.2.1 Biochemical properties

To annotate the wild‐type (WT) and mutant amino acids, all 20 amino acids were assigned to one of the five groups based on their biochemical properties, including hydrophobic (A, F, I, L, M, V, W, and Y), polar (N, Q, S, and T), negatively charged (D and E), positively charged (H, K, and R), and special (C, G, and P). The properties from AAindex 1 (Kawashima et al., 1999) and three basic properties, isoelectric point (pI), molecular weight, and molecular volume (*Annu Rev Biophys Bioeng*, 1984; Zamyatnin, 1972), were also included to describe both the WT and mutant residues.

To annotate the mutations, the biochemical scores from multiple amino acid substitution matrices and the statistical interpretations of protein contact potentials from the biochemical databases, AAindex 2 and 3 (Kawashima et al., 1999), were extracted based on the amino acid substitutions. To consider the effect on position‐specific conservation, scores were extracted from the Position Specific Substitution Matrix generated by PSI‐BLAST (Altschul et al., 1997; Camacho et al., 2009) against *nr* databases (Sayers et al., 2022) with three iterations.

To further consider the neighboring environment at the mutation site, the difference of the properties from AAindex 1 between WT and mutant amino acid was calculated, whose idea originated from previous work (Kulandaisamy et al., 2023). A sliding window of one, three, five, and seven residues at the mutation site was considered. WT properties were the average properties of the amino acids inside the window, while the mutant properties were unprocessed and extracted from the resources. This could consider the changes in biochemical properties between the mutant and the surrounding WT. Both the raw difference and the one normalized by the length of the protein sequence were included as features.

#### 4.2.2 Protein biophysical properties

The mutation effect on protein stability was computed by a sequence‐based method, SAAFEC‐SEQ (Li et al., 2021), and three structure‐based methods, mCSM‐Stability (Pires et al., 2014), DynaMut2 (Rodrigues et al., 2021), and DDMut (Zhou et al., 2023), using default settings. The reliability of these methods on AlphaFold2 models has been systematically studied in a previous work (Pan et al., 2022). All the calculations were measured using ΔΔG (Kcal/mol), with zero as a cutoff (ΔΔG >0: stabilizing; ΔΔG <0: destabilizing; and ΔΔG = 0: neutral).

#### 4.2.3 Local residue environment

The local environment at the mutation site was described from three aspects, namely neighboring amino acid frequency, general structural properties, and the residue contacts and atomic distance pattern, to identify local differences between neutral and disease‐causing mutations.

##### Neighboring amino acid frequency

To model the sequence‐based residue environment, the neighboring amino acid frequency was computed by considering a 15‐residue window at the mutation site (Kulandaisamy et al., 2023). All 20 amino acids were assigned into another seven different groups based on previous works (Caldararu et al., 2020), namely hydrophobic (A, L, and M), aliphatic (I and V), aromatic (F, Y, and W), long polar (E, Q, K, and R), short polar (H, S, T, and C), short charged/polar (D and N), and structure‐breaking (G and P). The count of amino acids in each group of the seven leading and following residues was computed and normalized using the length of the protein sequence to generate the frequency. This feature could be used to represent the biochemical property of the mutation environment.

##### General structural properties

Relative solvent accessibility (RSA) and residue depth were measured via Biopython packages (Cock et al., 2009) to quantify solvent exposure in the mutation site. Secondary structure and torsion angles of peptide bonds (phi and psi) were computed using the DSSP program (Joosten et al., 2011; Kabsch & Sander, 1983). To quantify the disorderliness of the protein structure, both predictions of IUPred (Dosztanyi, 2018; Erdos et al., 2021) and the pLDDT scores from the AlphaFold2 model were introduced.

##### Residue contacts and atomic distance pattern

Local residue contact at the mutation site of both WT and mutant structures (generated by MODELLER Marti‐Renom et al., 2000; Sali & Blundell, 1993; Webb & Sali, 2016) was computed using Arpeggio packages (Jubb et al., 2017). The atomic distance pattern at the mutation site was captured by graph‐based signatures (Pires et al., 2014). In graph‐based signatures, atoms with eight different pharmacophores are modeled as nodes and their contacts within a certain distance cutoff are modeled as edges. The residue environmental context is described using a cumulative distribution of different types of contacts via different configurations of distance steps and cutoffs from the mutation site.

#### 4.2.4 Functionality assessment

Two conventional variant effect predictors, Sorting Intolerant From Tolerant (SIFT) (Ng & Henikoff, 2003) and SNAP2 (Hecht et al., 2015), were used to estimate the impact of mutations on protein function based on conservation.

### 4.3 Machine learning analysis

To build the generalized model including all mutations in different proteins, the primary dataset was first divided into training (60%), validation (20%), and blind test (20%) sets, ensuring non‐redundancy between data sets according to unique protein position. Seven machine learning algorithms, including *Decision Trees*, *Random Forest*, *Extra Trees*, *Adaptive Boosting*, *Gradient Boosting*, *Extreme Gradient Boosting*, and *Logistic Regression*, were trained and tested on the training and validation sets first (Table S7), followed by the optimization using greedy feature selection (Figure S4A), whose algorithm has been well described in previous works (Myung et al., 2020; Rodrigues et al., 2021). After hyperparameter tuning using *HalvingRandomSearch*, three boosting‐based and tree‐based parameters were set, namely *n_estimators* = 80, *learning_rate* = 0.1, and *max_depth* = 7 (Figure S4B, Supplementary File halving_search.csv). Predictive performance was evaluated using accuracy, balanced accuracy (BACC), recall, precision, F1‐score, MCC, and area under the receiver operating characteristic curve (AUC).

To further enhance the capability of identifying AD‐causing mutations at the protein‐specific levels, the sample weights were tuned during the training process for 21 AD‐related proteins (Fang et al., 2022). Specifically, mutations from the target protein were assigned with weights = 11.0, while the mutations from non‐target proteins were assigned with weights = 1.0, by setting the “sample weights” of the training process in the Scikit‐Learn packages. This choice of weight was generated by optimizing the generalized performance. Protein‐specific performance was evaluated using accuracy, as not all the target proteins had both neutral and disease‐causing mutations.

Predictive performance of both our enhanced models was compared with different state‐of‐the‐art methods, including an AD‐specific method, Alz‐Disc (Kulandaisamy et al., 2023), two DMS‐based methods, namely DeMaSk (Munro & Singh, 2021) and Envision (Gray et al., 2018), three conventional predictors, including SIFT (Ng & Henikoff, 2003), SNAP2 (Hecht et al., 2015), and PROVEAN (Choi & Chan, 2015), and three deep learning‐based methods, which are AlphaMissense (Cheng et al., 2023), ESM1b (Brandes et al., 2023), and CPT‐1 (Jagota et al., 2023), respectively. A cutoff was used to convert the fitness score into disease‐causing and neutral phenotypes. The suggested cutoffs of 0.05, 0.0, and −2.5 were used for SIFT, SNAP2, and PROVEAN, respectively. In terms of the DMS‐based and deep learning‐based methods, we used the training set to determine a cutoff to maximize the performance of MCC, which has been described in previous work (Pan et al., 2023), and the final cutoffs used in this work were illustrated here (DeMaSk: −0.22, Envision: 0.97, AlphaMissense: 0.61, ESM1b: −8.18, CPT‐1: 0.39).

### 4.4 Web server

AlzDiscovery has been implemented as a user‐friendly interactive web server freely available to the research community at: https://biosig.lab.uq.edu.au/alzdiscovery/. Users can browse the effects of pathogenicity of all the possible missense mutations of 21 AD‐related proteins by choosing the mutation of interest. Detailed structure information at the mutation site, such as interatomic interactions, is displayed on the 3D viewers (Rose et al., 2018). Predicted phenotypes of saturation mutagenesis using our best performing models at the protein‐specific levels can be downloaded from the website for further analysis and patient monitoring.

## AUTHOR CONTRIBUTIONS

**Qisheng Pan:** Methodology; formal analysis; writing – original draft; visualization; validation. **Georgina Becerra Parra:** Methodology; data curation; formal analysis; writing – review and editing. **Yoochan Myung:** Software; writing – review and editing. **Stephanie Portelli:** Methodology; formal analysis; writing – review and editing. **Thanh Binh Nguyen:** Methodology; formal analysis; writing – review and editing. **David B. Ascher:** Methodology; writing – review and editing; supervision; conceptualization; formal analysis.

## CONFLICT OF INTEREST STATEMENT

The authors declared no conflict of interest.

## Supporting information


**Appendix S1.** Introduction of 21 AD‐related proteins and their mechanism toward AD (Table S1). Distributions of mutation data in the primary dataset (Table S2) and validation set (Table S3). Performance of development of the initial generic models and enhanced models after weight tuning (Table S4, Table S7, Figure S1, Figure S4). Performance of the leave‐one‐protein‐out evaluation (Table S5). Comparison of performance on the protein‐specific level between AlzDiscovery and Alz‐Disc in the independent validation (Table S6). Mutation landscape leading to AD of three strong risk factors, APOE, MAPT, and TREM2 (Figure S2). Analysis of intramolecular residue contacts of three mutations in APP, PSEN1, and PSEN2, respectively (Figure S3).
**Appendix S2.** Primary dataset in Excel spreadsheet. Independent validation set in Excel spreadsheet; All AlphaFold2 models analyzed in this work. Summary of *HalvingRandomSearch* of hyperparameter tuning in Excel spreadsheet.
