# Supplementary material for: AlzDiscovery: A computational tool to identify Alzheimer's disease‐causing missense mutations using protein structure information
Source: Protein Sci. 2024 Sep 14;33(10):e5147. doi: 10.1002/pro.5147 (PMC11401060; doi:10.1002/pro.5147)
Supplement: Supplementary file 1 — Appendix S1. Introduction of 21 AD‐related proteins and their mechanism toward AD (Table S1). Distributions of mutation data in the primary dataset (Table S2) and validation set (Table S3). Performance of development of the initial generic models and enhanced models after weight tuning (Table S4, Table S7, Figure S1, Figure S4). Performance of the leave‐one‐protein‐out evaluation (Table S5). Comparison of performance on the protein‐specific level between AlzDiscovery and Alz‐Disc in the independent validation (Table S6). Mutation landscape leading to AD of three strong risk factors, APOE, MAPT, and TREM2 (Figure S2). Analysis of intramolecular residue contacts of three mutations in APP, PSEN1, and PSEN2, respectively (Figure S3). Appendix S2. Primary dataset in Excel spreadsheet. Independent validation set in Excel spreadsheet; All AlphaFold2 models analyzed in this work. Summary of HalvingRandomSearch of hyperparameter tuning in Excel spreadsheet. [file PRO-33-e5147-s001.pdf]

# Supporting Information

# AlzDiscovery: a computational tool to identify Alzheimer's Disease-causing missense mutations using protein structure information

Qisheng Pan[1,2], Georgina Becerra Parra[1,2], Yoochan Myung[1,2], Stephanie Portelli[1,2], Thanh Binh Nguyen[1,2], David B. Ascher[1,2,*]

[1]The Australian Centre for Ecogenomics, School of Chemistry and Molecular Bioscience, University of Queensland, Brisbane Queensland 4072, Australia

[2]Computational Biology and Clinical Informatics, Baker Heart and Diabetes Institute, Melbourne Victoria 3004, Australia

*To whom correspondence should be addressed to D.B.A. Tel: +61 7 336 53991; Email: d.ascher@uq.edu.au.

# TABLES

**Table S1.** A short introduction of each of the 21 Alzheimer's Disease (AD)-related proteins

| Gene symbol | UniProt ID | Protein name | Association with AD | Introduction |
|---|---|---|---|---|
| APP | P05067 | Amyloid Precursor Protein | Aβ generation / familial AD | APP could be cleavaged by β-secretase and γ-secretase and produce the Aβ peptides with 39-43 amino acids in length, which are the major components of the neuritic plaques (26). APP is strongly associated with the Early-onset AD (EOAD) and familial AD (fAD), and the mutations in APP will result in the increase of the Aβ42/Aβ40 ratio, bringing a stronger neural toxicity (39). |
| PSEN1 | P49768 | Presenilin 1 | Aβ generation / familial AD | The PSEN1 and 2 proteins are the major components of γ-secretase which participates in the cleavage of APP and produces the Aβ peptides. Loss-of-function mutations in PSEN1 protein are suspected to increase the ratio of Aβ42/Aβ40 (24), which could produce more neuritic plaques with stronger damaging effects. |
| PSEN2 | P49810 | Presenilin 2 | Aβ generation / familial AD | The PSEN 1 and 2 proteins are the major components of γ-secretase which participates in the cleavage of APP and produce the Aβ peptides. Mutations in PSEN2 are suspected to increase |

| | | | | |
|---|---|---|---|---|
| | | | | the activity of γ-secretase (6). |
| A2M | P01023 | Alpha-2 Macroglobulin | Aβ related | A2M, a major component of the innate immune system (41), is reported to mediate the clearance and degradation of β-amyloid (Aβ) peptides (3). |
| PLAU | P00749 | Urokinase-type plasminogen activator | Aβ related | PLAU gene encodes the Urokinase-type plasminogen activator (uPA), which plays a role in the breakdown of APP and the degradation of Aβ (15). |
| PRNP | P04156 | Prion protein | Aβ related | PRNP is associated with the pathogenesis of AD by interacting the β-secretease BACE1 and the cleavage of Alzheimer's APP (34; 28). |
| Alternative PRNP | F7VJQ1 | Alternative prion protein | Aβ related | Alternative PRNP has a completely different amino acid sequence from PRNP due to an alternative open reading frame (40), and it is coexpressed with prion protein in human neurons. |
| ABCA7 | Q8IZY2 | ATP-Binding Cassette transporter A7 | Aβ related / Lipid biology | ABCA7, expressed by both neuron and microglia cells in the human brain, regulates lipid metabolism (32) and promotes the clearance of Aβ (17). The variants of ABCA7 could increase the risk of AD from 80% to 400% reported in Africans and European ancestry populations (10; 2; 13). |

| | | | | |
|---|---|---|---|---|
| CLU | P10909 | Clusterin | Aβ related / Lipid biology | CLU was identified as a LOAD risk factor in the Genome-wide Association Study (GWAS) (21; 27). Its function is related to the alteration of Aβ aggregation and promotes the clearance of Aβ (16). |
| ADAM10 | O14672 | A Disintegrin And Metalloproteinase 10 | Aβ related / tau pathology | ADAM10 protein plays a role in the regulation of α-secretase and the cleavage of APP (14; 35). ADAM10 can also potentially affect pathology of AD via reducing tau pathology, regulating normal synaptic functions, and enhancing hippocampal neurogenesis and the homeostasis of neuronal networks (43). |
| APOE | P02649 | Apolipoprotein E | Lipid biology | APOE lipoprotein participates in the lipid metabolism by binding to a number of cell surface receptors (25). Three polymorphic alleles, ε2, ε3 and ε4, are reported with a worldwide frequency of around 8%, 78%, and 14%. Among them, the ε4-APOE could drastically increase the risk of Late-onset AD (LOAD). |
| MAPT | P10636 | Microtubule Associated Protein Tau | Tau pathology | The main function of tau protein is to stabilise the microtubule (5). The hyperphosphorylated tau will detach from the microtubule, resulting in the destabilisation of cellular skeleton and the aggregation of the neurofibrillary tangles (1). |
| UNC5C | O95185 | Unc-5 netrin | Tau pathology | UNC5C may induce the activation of death-associated protein kinase 1 (DAPK1), a new |

| | | | | |
|---|---|---|---|---|
| | | receptor C | | component involved in AD pathogenesis and the aberrant tau (29). The genetic variants of UNC5C are also reported to increase the risk of AD (42). |
| VCP | P55072 | Valosin-Containing Protein | Tau pathology | VCP, ubiquitously expressed in human cells, plays a role in protein degradation through ubiquitin proteasome-based system and autophagy pathways. Recent study suggested its relationship with tau pathology (36). |
| MT-ND1 | P03886 | Mitochondrial encoded ND1 protein | Oxidative stress | MT-ND1 and MT-ND2 modulates the oxidative phosphorylation in human cells (31). Some studies also suggested the involvement of the mutations in mitochondrial DNA in AD pathology (20). |
| MT-ND2 | P03891 | Mitochondrial encoded ND2 protein | Oxidative stress | Described as MT-ND1. |
| NOS3 | P29474 | Nitric Oxide Synthase-3 | Oxidative stress | The increased expression of NOS3 is linked to neural degeneration in AD brains. Dysfunction of NOS3 could induce the increment of cellular reactive oxygen species, and damage the CNS (11; 12). |
| TF | P02787 | Transferrin | Oxidative | TF was suggested to be a candidate locus of AD because of its functions on iron |

| | | | | |
|---|---|---|---|---|
| | | | stress | transportation and the modulation of free radical generation (23). The relationship between iron metabolism and AD gets an increasing attention (9). |
| CSF1R | P07333 | Colony-Stimulating Factor 1 Receptor | Innate immunity | CFS1R is a key tyrosine kinase transmembrane receptor maintaining the microglia homeostasis (8), neurogenesis, and neuronal survival in the Central Nervous System (CNS) (33; 22). Recent works suggested the association of its variants with AD (4; 37; 18). |
| TREM2 | Q9NZC2 | Triggering Receptor Expressed on Myeloid cells 2 | Innate immunity | TREM2 protein is a new biomarker of AD proposed recently as the LOF mutations cause 4.9-fold increased risk of AD (30). In the human brain, TREM2 is exclusively expressed in the microglia. It could interact with various proteins and ligands, such as APOE and Aβ, and activate the signalling pathway through DNAX-activation protein 12 (DAP12) (38; 19). |
| PCDH11X | Q9BZA7 | Protocadherin 11 X-linked protein | Others | PCDH11X was suggested to be one of the risk factors leading to LOAD by a two-stage GWAS analysis (7). It is suspected to play a functional role in cell-cell interaction in the CNS. |

**Table S2.** Distribution of mutations from 21 Alzheimer's Disease (AD)-related proteins in the primary dataset

| Gene | Sequence length | # mutations | # Disease-causing | # Neutral |
|---|---|---|---|---|
| A2M | 1474 | 8 | 0 | 8 |
| ABCA7 | 2146 | 41 | 1 | 40 |
| ADAM10 | 748 | 2 | 2 | 0 |
| APOE | 317 | 24 | 8 | 16 |
| APP | 770 | 57 | 28 | 29 |
| CLU | 449 | 7 | 0 | 7 |
| CSF1R | 972 | 39 | 2 | 37 |
| MAPT | 758 | 38 | 3 | 35 |
| MT-ND1 | 318 | 47 | 1 | 46 |
| MT-ND2 | 347 | 19 | 1 | 18 |
| NOS3 | 1203 | 19 | 0 | 19 |
| PCDH11X | 1347 | 8 | 0 | 8 |
| PLAU | 431 | 16 | 0 | 16 |
| Alternative PRNP | 73 | 7 | 0 | 7 |
| PRNP | 253 | 11 | 0 | 11 |
| PSEN1 | 467 | 253 | 250 | 3 |

| | | | | |
|---|---|---|---|---|
| PSEN2 | 448 | 36 | 16 | 20 |
| TF | 698 | 20 | 0 | 20 |
| TREM2 | 230 | 21 | 6 | 15 |
| UNC5C | 931 | 4 | 1 | 3 |
| VCP | 806 | 3 | 1 | 2 |
| Total | / | 680 | 320 | 360 |

**Table S3.** Distribution of mutations of the clinical validation

| Gene | # mutations | # Disease-causing | # Neutral |
|---|---|---|---|
| ADAM10 | 2 | 0 | 2 |
| APOE | 8 | 0 | 8 |
| APP | 5 | 5 | 0 |
| CSF1R | 13 | 2 | 11 |
| MAPT | 30 | 0 | 30 |
| PRNP | 22 | 0 | 22 |
| PSEN1 | 2 | 1 | 1 |
| PSEN2 | 5 | 4 | 1 |
| TF | 5 | 0 | 5 |
| TREM2 | 3 | 3 | 0 |
| VCP | 30 | 0 | 30 |
| Total | 125 | 15 | 110 |

**Table S4.** Overall performance of AlzDiscovery using different training weights

| Weights | Test | BACC* | F1-score | MCC* | Recall | Precision | AUC* |
|---|---|---|---|---|---|---|---|
| 1 | 10-CV | 0.89 | 0.89 | 0.79 | 0.86 | 0.92 | 0.94 |
| 11 | 10-CV | 0.89 | 0.88 | 0.79 | 0.86 | 0.91 | 0.95 |
| 1 | Blind test | 0.87 | 0.87 | 0.75 | 0.86 | 0.88 | 0.94 |
| 11 | Blind test | 0.90 | 0.89 | 0.79 | 0.88 | 0.90 | 0.95 |

*BACC represents Balanced Accuracy, MCC is Matthew's Correlation Coefficient, and AUC represents the Aera Under the Receiver Operating Characteristics (ROC) Curve.

**Table S5.** Performance of leave-one-protein-out evaluation

| Gene | Accuracy | BACC | F1-score | MCC | Recall | Precision | AUC |
|---|---|---|---|---|---|---|---|
| A2M | 1 | / | / | / | / | / | / |
| ABCA7 | 0.98 | / | / | / | / | / | / |
| ADAM10 | 0 | / | / | / | / | / | / |
| Alternative PRNP | 1 | / | / | / | / | / | / |
| APOE | 0.71 | / | / | / | / | / | / |
| APP | 0.74 | / | / | / | / | / | / |
| CLU | 1 | / | / | / | / | / | / |
| CSF1R | 0.83 | / | / | / | / | / | / |
| MAPT | 0.89 | / | / | / | / | / | / |
| MT-ND1 | 0.79 | / | / | / | / | / | / |
| MT-ND2 | 0.84 | / | / | / | / | / | / |
| NOS3 | 0.89 | / | / | / | / | / | / |
| PCDH11X | 1 | / | / | / | / | / | / |
| PLAU | 0.75 | / | / | / | / | / | / |
| PRNP | 0.73 | / | / | / | / | / | / |
| PSEN1 | 0.21 | / | / | / | / | / | / |

| | | | | | | | |
|---|---|---|---|---|---|---|---|
| PSEN2 | 0.69 | / | / | / | / | / | / |
| TF | 0.9 | / | / | / | / | / | / |
| TREM2 | 0.81 | / | / | / | / | / | / |
| UNC5C | 1 | / | / | / | / | / | / |
| VCP | 0.67 | / | / | / | / | / | / |
| Overall | 0.59 | 0.57 | 0.37 | 0.19 | 0.26 | 0.67 | 0.65 |

**Table S6.** Protein-specific performance of AlzDiscovery and Alz-Disc on clinical validation set

| Gene | Accuracy | |
|---|---|---|
| | AlzDiscovery | Alz-Disc |
| ADAM10 | 1.00 | 1.00 |
| APOE | 1.00 | 0.75 |
| APP | 0.60 | 0.20 |
| CSF1R | 0.85 | 0.85 |
| MAPT | 1.00 | 0.57 |
| PRNP | 0.73 | 0.95 |
| PSEN1 | 0.50 | 0.50 |
| PSEN2 | 0.40 | 0.60 |
| TF | 1.00 | 1.00 |
| TREM2 | 0.33 | 0.00 |
| VCP | 0.87 | 1.00 |

**Table S7.** Performance of initial generic model using different machine learning algorithms

| Algorithm | 10-fold Cross Validation on training and validation set* (MCC) | validation set* (MCC) |
|---|---|---|
| Adaptive Boosting | 0.72 | 0.68 |
| Decision Trees | 0.52 | 0.68 |
| Extra Trees | 0.59 | 0.64 |
| **Gradient Boosting** | **0.74** | **0.77** |
| Logistic Regression | 0.58 | 0.59 |
| Random Forest | 0.59 | 0.70 |
| Extreme Gradient Boosting | 0.68 | 0.77 |

*Primary dataset was first divided into training (60%), validation (20%), and blind test (20%) set. For model development, the data only from training and validation were used.

## FIGURES

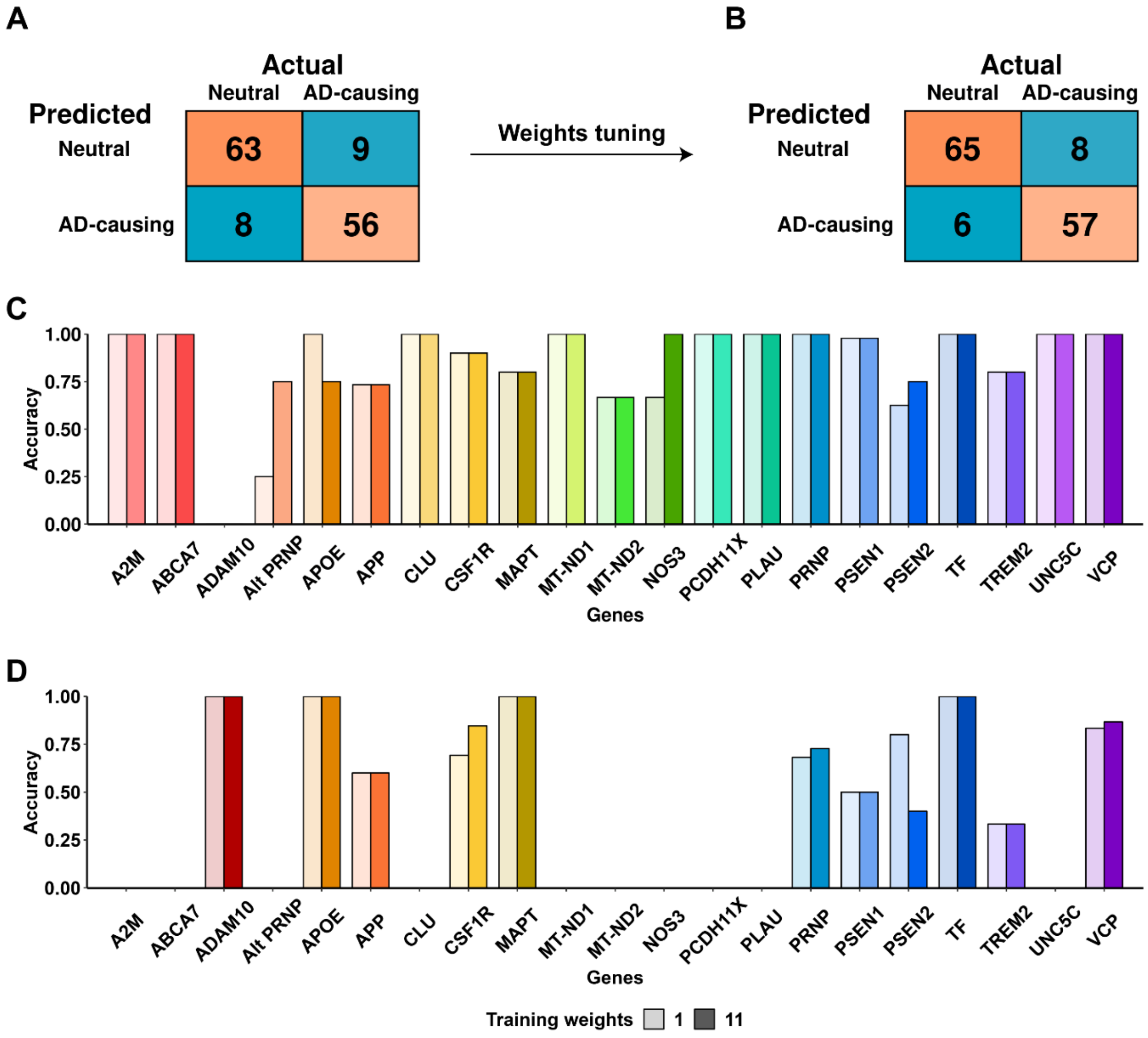


**Figure S1.** Performance comparison of two models with different weights for the training process. More correctly predicted samples were obtained from the model with weight=11 on the target protein (B), compared with the confusion matrix from the original model (A). Protein-specific performance of blind test (C) and clinical validation (D) was evaluated using accuracy due to the imbalanced distribution of mutation labels. Performance on ADAM10 of the blind test was 0 due to a small number of mutations (n=2) in the dataset (C). The missing performance in clinical validation (D) was due to the difference of target proteins in the validation set.

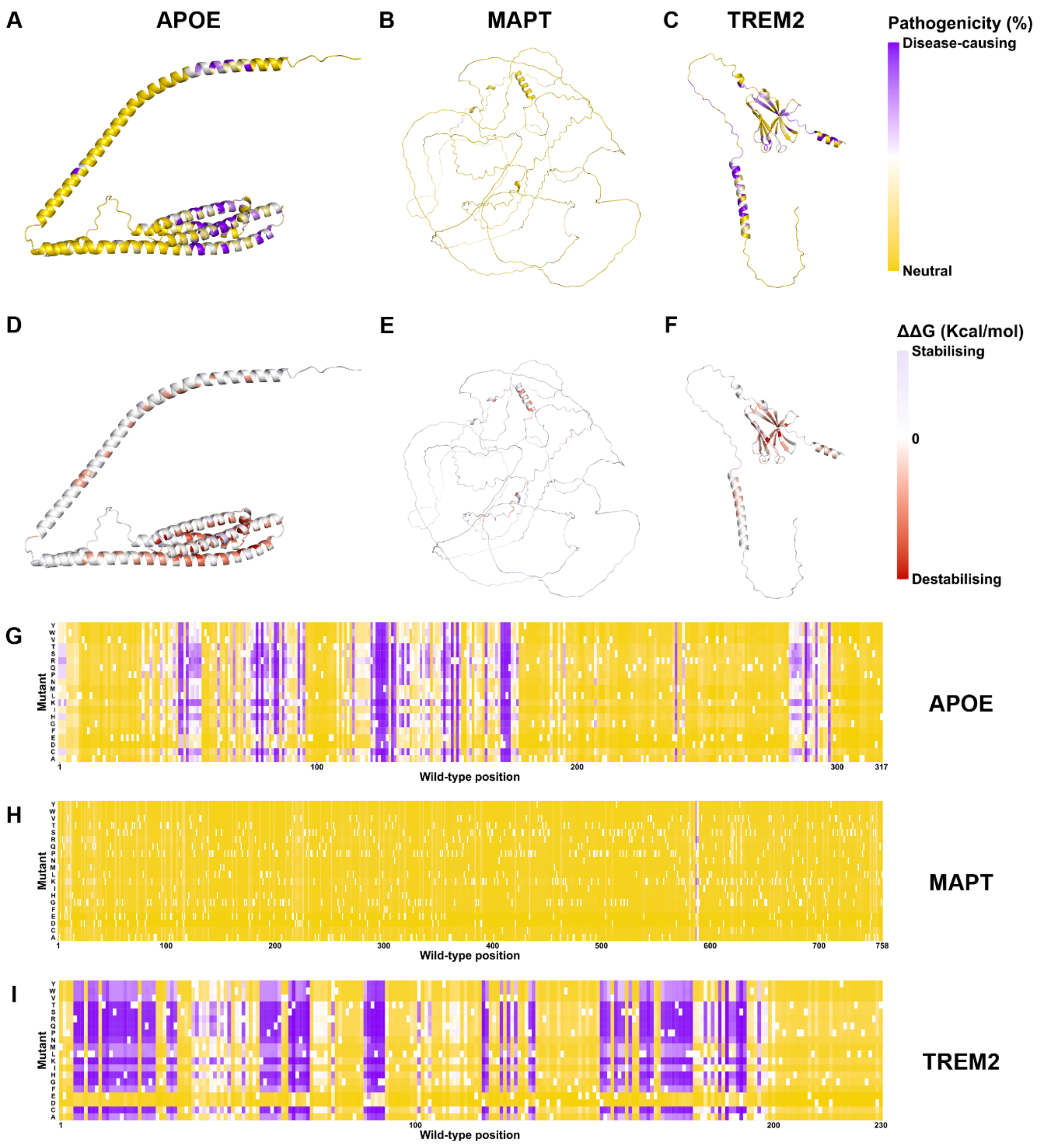


**Figure S2.** Mutation landscape leading to AD of three strong risk factors, APOE, MAPT, and TREM2. Protein structures are coloured based on two colour schemes, namely the average probability leading to diseases of all possible missense mutations at each mutation site (A-C) and the change of protein stability upon mutations at each mutation site (D-F), while the heatmaps present detailed results of different mutants at each site of three proteins (G-I).

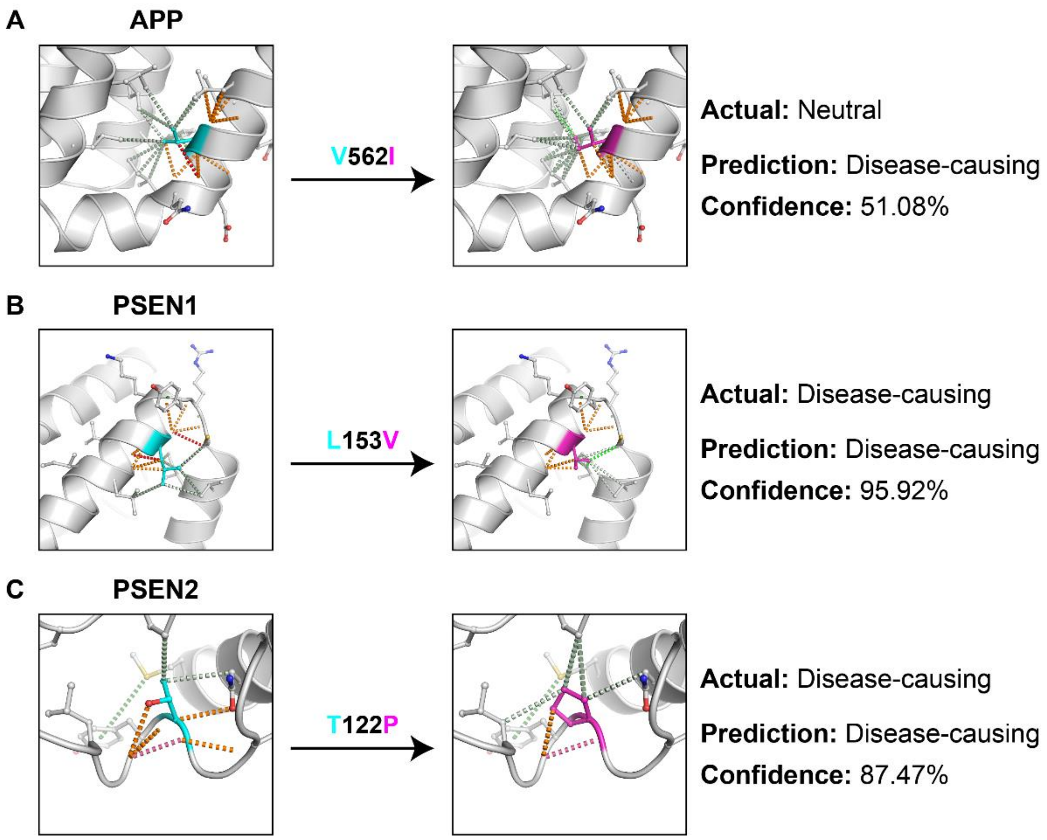


**Figure S3.** Structural analysis on three mutations in the familial AD-related proteins, namely APP (A), PSEN1 (B), and PSEN2 (C). Intramolecular residue contacts were generated using Arpeggio [cite 27964945]. The actual and predictive phenotypes with predictive confidence were listed on the right column.

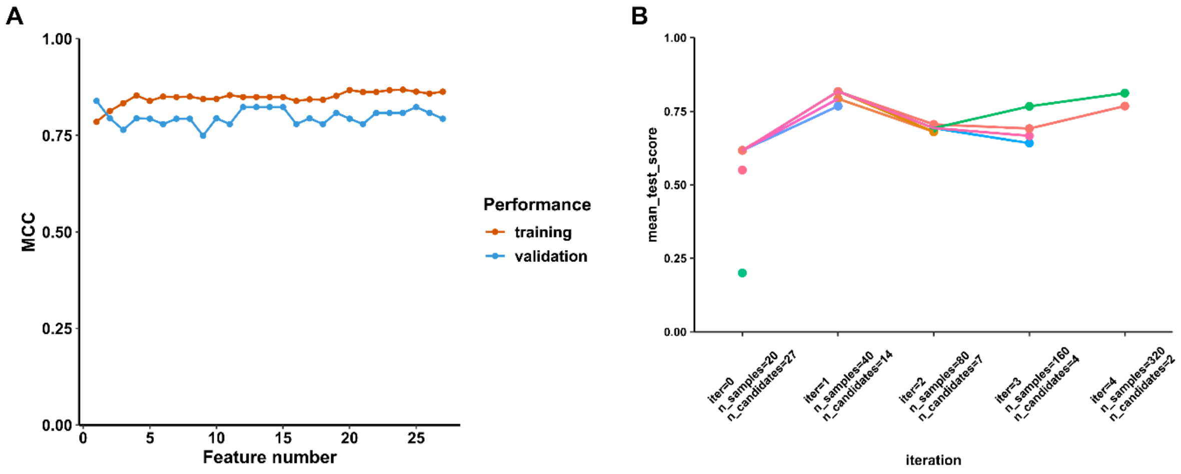


**Figure S4.** Optimisation process of the development of machine learning models. Greedy feature selection was used to simplify the complexity of model (A), followed by the hyperparameter tunning using *HalvingRandomSearch* in sci-kit learn packages (B).

## REFERENCE


1. Ashrafian H, Zadeh EH, Khan RH (2021) Review on Alzheimer's disease: Inhibition of amyloid beta and tau tangle formation. Int J Biol Macromol 167:382-394. PMID: 33278431 {Medline}
2. Bellenguez C, Charbonnier C, Grenier-Boley B, Quenez O, Le Guennec K, Nicolas G, Chauhan G, Wallon D, Rousseau S, Richard AC, Boland A, Bourque G, Munter HM, Olaso R, Meyer V, Rollin-Sillaire A, Pasquier F, Letenneur L, Redon R, Dartigues JF, Tzourio C, Frebourg T, Lathrop M, Deleuze JF, Hannequin D, Genin E, Amouyel P, Debette S, Lambert JC, Campion D, collaborators CM (2017) Contribution to Alzheimer's disease risk of rare variants in TREM2, SORL1, and ABCA7 in 1779 cases and 1273 controls. Neurobiol Aging 59:220 e221-220 e229. PMID: 28789839 {Medline}
3. Blacker D, Wilcox MA, Laird NM, Rodes L, Horvath SM, Go RC, Perry R, Watson B, Jr., Bassett SS, McInnis MG, Albert MS, Hyman BT, Tanzi RE (1998) Alpha-2 macroglobulin is genetically associated with Alzheimer disease. Nat Genet 19:357-360. PMID: 9697696 {Medline}
4. Blue EE, Bis JC, Dorschner MO, Tsuang DW, Barral SM, Beecham G, Below JE, Bush WS, Butkiewicz M, Cruchaga C, DeStefano A, Farrer LA, Goate A, Haines J, Jaworski J, Jun G, Kunkle B, Kuzma A, Lee JJ, Lunetta KL, Ma Y, Martin E, Naj A, Nato AQ, Navas P, Nguyen H, Reitz C, Reyes D, Salerno W, Schellenberg GD, Seshadri S, Sohi H, Thornton TA, Valadares O, van Duijn C, Vardarajan BN, Wang LS, Boerwinkle E, Dupuis J, Pericak-Vance MA, Mayeux R, Wijsman EM, on behalf of the Alzheimer's Disease Sequencing P (2018) Genetic Variation in Genes Underlying Diverse Dementias May Explain a Small Proportion of Cases in the Alzheimer's Disease Sequencing Project. Dement Geriatr Cogn Disord 45:1-17. PMID: 29486463 {Medline}

5. Boyarko B, Hook V (2021) Human Tau Isoforms and Proteolysis for Production of Toxic Tau Fragments in Neurodegeneration. Front Neurosci 15:702788. PMID: 34744602 {Medline}

6. Cai Y, An SS, Kim S (2015) Mutations in presenilin 2 and its implications in Alzheimer's disease and other dementia-associated disorders. Clin Interv Aging 10:1163-1172. PMID: 26203236 {Medline}

7. Carrasquillo MM, Zou F, Pankratz VS, Wilcox SL, Ma L, Walker LP, Younkin SG, Younkin CS, Younkin LH, Bisceglio GD, Ertekin-Taner N, Crook JE, Dickson DW, Petersen RC, Graff-Radford NR, Younkin SG (2009) Genetic variation in PCDH11X is associated with susceptibility to late-onset Alzheimer's disease. Nat Genet 41:192-198. PMID: 19136949 {Medline}

8. Chitu V, Biundo F, Shlager GGL, Park ES, Wang P, Gulinello ME, Gokhan S, Ketchum HC, Saha K, DeTure MA, Dickson DW, Wszolek ZK, Zheng D, Croxford AL, Becher B, Sun D, Mehler MF, Stanley ER (2020) Microglial Homeostasis Requires Balanced CSF-1/CSF-2 Receptor Signaling. Cell Rep 30:3004-3019 e3005. PMID: 32130903 {Medline}

9. Chung JY, Kim HS, Song J (2018) Iron metabolism in diabetes-induced Alzheimer's disease: a focus on insulin resistance in the brain. Biometals 31:705-714. PMID: 30043289 {Medline}

10. Cukier HN, Kunkle BW, Vardarajan BN, Rolati S, Hamilton-Nelson KL, Kohli MA, Whitehead PL, Dombroski BA, Van Booven D, Lang R, Dykxhoorn DM, Farrer LA, Cuccaro ML, Vance JM, Gilbert JR, Beecham GW, Martin ER, Carney RM, Mayeux R, Schellenberg GD, Byrd GS, Haines JL, Pericak-Vance MA, Alzheimer's Disease Genetics C (2016) ABCA7 frameshift deletion associated with Alzheimer disease in African Americans. Neurol Genet 2:e79. PMID: 27231719 {Medline}

11. Dahiyat M, Cumming A, Harrington C, Wischik C, Xuereb J, Corrigan F, Breen G, Shaw D, St Clair D (1999) Association between Alzheimer's disease and the NOS3 gene. Ann Neurol 46:664-667. PMID: 10514107 {Medline}

12. de la Monte SM, Chiche J, von dem Bussche A, Sanyal S, Lahousse SA, Janssens SP, Bloch KD (2003) Nitric oxide synthase-3 overexpression causes apoptosis and impairs neuronal mitochondrial function: relevance to Alzheimer's-type neurodegeneration. Lab Invest 83:287-298. PMID: 12594242 {Medline}
13. De Roeck A, Van den Bossche T, van der Zee J, Verheijen J, De Coster W, Van Dongen J, Dillen L, Baradaran-Heravi Y, Heeman B, Sanchez-Valle R, Llado A, Nacmias B, Sorbi S, Gelpi E, Grau-Rivera O, Gomez-Tortosa E, Pastor P, Ortega-Cubero S, Pastor MA, Graff C, Thonberg H, Benussi L, Ghidoni R, Binetti G, de Mendonca A, Martins M, Borroni B, Padovani A, Almeida MR, Santana I, Diehl-Schmid J, Alexopoulos P, Clarimon J, Lleo A, Fortea J, Tsolaki M, Koutroumani M, Matej R, Rohan Z, De Deyn P, Engelborghs S, Cras P, Van Broeckhoven C, Sleegers K, European Early-Onset Dementia c (2017) Deleterious ABCA7 mutations and transcript rescue mechanisms in early onset Alzheimer's disease. Acta Neuropathol 134:475-487. PMID: 28447221 {Medline}
14. Endres K, Deller T (2017) Regulation of Alpha-Secretase ADAM10 In vitro and In vivo: Genetic, Epigenetic, and Protein-Based Mechanisms. Front Mol Neurosci 10:56. PMID: 28367112 {Medline}
15. Finckh U, van Hadeln K, Muller-Thomsen T, Alberici A, Binetti G, Hock C, Nitsch RM, Stoppe G, Reiss J, Gal A (2003) Association of late-onset Alzheimer disease with a genotype of PLAU, the gene encoding urokinase-type plasminogen activator on chromosome 10q22.2. Neurogenetics 4:213-217. PMID: 12898287 {Medline}
16. Foster EM, Dangla-Valls A, Lovestone S, Ribe EM, Buckley NJ (2019) Clusterin in Alzheimer's Disease: Mechanisms, Genetics, and Lessons From Other Pathologies. Front Neurosci 13:164. PMID: 30872998 {Medline}
17. Fu Y, Hsiao JH, Paxinos G, Halliday GM, Kim WS (2016) ABCA7 Mediates Phagocytic Clearance of Amyloid-beta in the Brain. J Alzheimers Dis 54:569-584. PMID: 27472885 {Medline}

18. Giau VV, Senanarong V, Bagyinszky E, An SSA, Kim S (2019) Analysis of 50 Neurodegenerative Genes in Clinically Diagnosed Early-Onset Alzheimer's Disease. Int J Mol Sci 20. PMID: 30917570 {Medline}

19. Gratuze M, Leyns CEG, Holtzman DM (2018) New insights into the role of TREM2 in Alzheimer's disease. Mol Neurodegener 13:66. PMID: 30572908 {Medline}

20. Grazina M, Pratas J, Silva F, Oliveira S, Santana I, Oliveira C (2006) Genetic basis of Alzheimer's dementia: role of mtDNA mutations. Genes Brain Behav 5 Suppl 2:92-107. PMID: 16681804 {Medline}

21. Hardy J, Singleton A (2009) Genomewide association studies and human disease. N Engl J Med 360:1759-1768. PMID: 19369657 {Medline}

22. Hu B, Duan S, Wang Z, Li X, Zhou Y, Zhang X, Zhang YW, Xu H, Zheng H (2021) Insights Into the Role of CSF1R in the Central Nervous System and Neurological Disorders. Front Aging Neurosci 13:789834. PMID: 34867307 {Medline}

23. Hussain RI, Ballard CG, Edwardson JA, Morris CM (2002) Transferrin gene polymorphism in Alzheimer's disease and dementia with Lewy bodies in humans. Neurosci Lett 317:13-16. PMID: 11750985 {Medline}

24. Kelleher RJ, 3rd, Shen J (2017) Presenilin-1 mutations and Alzheimer's disease. Proc Natl Acad Sci U S A 114:629-631. PMID: 28082723 {Medline}

25. Kim J, Castellano JM, Jiang H, Basak JM, Parsadanian M, Pham V, Mason SM, Paul SM, Holtzman DM (2009) Overexpression of low-density lipoprotein receptor in the brain markedly inhibits amyloid deposition and increases extracellular A beta clearance. Neuron 64:632-644. PMID: 20005821 {Medline}

26. Knopman DS, Amieva H, Petersen RC, Chetelat G, Holtzman DM, Hyman BT, Nixon RA, Jones DT (2021) Alzheimer disease. Nat Rev Dis Primers 7:33. PMID: 33986301 {Medline}

27. Lambert JC, Heath S, Even G, Campion D, Sleegers K, Hiltunen M, Combarros O, Zelenika D, Bullido MJ, Tavernier B, Letenneur L, Bettens K, Berr C, Pasquier F, Fievet N, Barberger-Gateau P, Engelborghs S, De Deyn P, Mateo I, Franck A, Helisalmi S,

Porcellini E, Hanon O, European Alzheimer's Disease Initiative I, de Pancorbo MM, Lendon C, Dufouil C, Jaillard C, Leveillard T, Alvarez V, Bosco P, Mancuso M, Panza F, Nacmias B, Bossu P, Piccardi P, Annoni G, Seripa D, Galimberti D, Hannequin D, Licastro F, Soininen H, Ritchie K, Blanche H, Dartigues JF, Tzourio C, Gut I, Van Broeckhoven C, Alperovitch A, Lathrop M, Amouyel P (2009) Genome-wide association study identifies variants at CLU and CR1 associated with Alzheimer's disease. Nat Genet 41:1094-1099. PMID: 19734903 {Medline}

28. Lauren J, Gimbel DA, Nygaard HB, Gilbert JW, Strittmatter SM (2009) Cellular prion protein mediates impairment of synaptic plasticity by amyloid-beta oligomers. Nature 457:1128-1132. PMID: 19242475 {Medline}

29. Li Q, Wang BL, Sun FR, Li JQ, Cao XP, Tan L (2018) The role of UNC5C in Alzheimer's disease. Ann Transl Med 6:178. PMID: 29951500 {Medline}

30. Li R, Wang X, He P (2021) The most prevalent rare coding variants of TREM2 conferring risk of Alzheimer's disease: A systematic review and meta-analysis. Exp Ther Med 21:347. PMID: 33732320 {Medline}

31. Lunnon K, Keohane A, Pidsley R, Newhouse S, Riddoch-Contreras J, Thubron EB, Devall M, Soininen H, Kloszewska I, Mecocci P, Tsolaki M, Vellas B, Schalkwyk L, Dobson R, Malik AN, Powell J, Lovestone S, Hodges A, AddNeuroMed C (2017) Mitochondrial genes are altered in blood early in Alzheimer's disease. Neurobiol Aging 53:36-47. PMID: 28208064 {Medline}

32. Lyssenko NN, Pratico D (2021) ABCA7 and the altered lipidostasis hypothesis of Alzheimer's disease. Alzheimers Dement 17:164-174. PMID: 33336544 {Medline}

33. Nandi S, Gokhan S, Dai XM, Wei S, Enikolopov G, Lin H, Mehler MF, Stanley ER (2012) The CSF-1 receptor ligands IL-34 and CSF-1 exhibit distinct developmental brain expression patterns and regulate neural progenitor cell maintenance and maturation. Dev Biol 367:100-113. PMID: 22542597 {Medline}

34. Parkin ET, Watt NT, Hussain I, Eckman EA, Eckman CB, Manson JC, Baybutt HN, Turner AJ, Hooper NM (2007) Cellular prion protein regulates beta-secretase cleavage of the

Alzheimer's amyloid precursor protein. Proc Natl Acad Sci U S A 104:11062-11067. PMID: 17573534 {Medline}

35. Pereira Vatanabe I, Peron R, Mantellatto Grigoli M, Pelucchi S, De Cesare G, Magalhaes T, Manzine PR, Figueredo Balthazar ML, Di Luca M, Marcello E, Cominetti MR (2021) ADAM10 Plasma and CSF Levels Are Increased in Mild Alzheimer's Disease. Int J Mol Sci 22. PMID: 33670873 {Medline}
36. Saha I, Yuste-Checa P, Da Silva Padilha M, Guo Q, Korner R, Holthusen H, Trinkaus VA, Dudanova I, Fernandez-Busnadiego R, Baumeister W, Sanders DW, Gautam S, Diamond MI, Hartl FU, Hipp MS (2023) The AAA+ chaperone VCP disaggregates Tau fibrils and generates aggregate seeds in a cellular system. Nat Commun 14:560. PMID: 36732333 {Medline}
37. Sassi C, Nalls MA, Ridge PG, Gibbs JR, Lupton MK, Troakes C, Lunnon K, Al-Sarraj S, Brown KS, Medway C, Lord J, Turton J, Bras J, Consortium A, Blumenau S, Thielke M, Josties C, Freyer D, Dietrich A, Hammer M, Baier M, Dirnagl U, Morgan K, Powell JF, Kauwe JS, Cruchaga C, Goate AM, Singleton AB, Guerreiro R, Hodges A, Hardy J (2018) Mendelian adult-onset leukodystrophy genes in Alzheimer's disease: critical influence of CSF1R and NOTCH3. Neurobiol Aging 66:179 e117-179 e129. PMID: 29544907 {Medline}
38. Takahashi K, Rochford CD, Neumann H (2005) Clearance of apoptotic neurons without inflammation by microglial triggering receptor expressed on myeloid cells-2. J Exp Med 201:647-657. PMID: 15728241 {Medline}
39. Tcw J, Goate AM (2017) Genetics of beta-Amyloid Precursor Protein in Alzheimer's Disease. Cold Spring Harb Perspect Med 7. PMID: 28003277 {Medline}
40. Vanderperre B, Staskevicius AB, Tremblay G, McCoy M, O'Neill MA, Cashman NR, Roucou X (2011) An overlapping reading frame in the PRNP gene encodes a novel polypeptide distinct from the prion protein. FASEB J 25:2373-2386. PMID: 21478263 {Medline}

41. Varma VR, Varma S, An Y, Hohman TJ, Seddighi S, Casanova R, Beri A, Dammer EB, Seyfried NT, Pletnikova O, Moghekar A, Wilson MR, Lah JJ, O'Brien RJ, Levey AI, Troncoso JC, Albert MS, Thambisetty M (2017) Alpha-2 macroglobulin in Alzheimer's disease: a marker of neuronal injury through the RCAN1 pathway. Mol Psychiatry 22:13-23. PMID: 27872486 {Medline}

42. Wetzel-Smith MK, Hunkapiller J, Bhangale TR, Srinivasan K, Maloney JA, Atwal JK, Sa SM, Yaylaoglu MB, Foreman O, Ortmann W, Rathore N, Hansen DV, Tessier-Lavigne M, Alzheimer's Disease Genetics C, Mayeux R, Pericak-Vance M, Haines J, Farrer LA, Schellenberg GD, Goate A, Behrens TW, Cruchaga C, Watts RJ, Graham RR (2014) A rare mutation in UNC5C predisposes to late-onset Alzheimer's disease and increases neuronal cell death. Nat Med 20:1452-1457. PMID: 25419706 {Medline}

43. Yuan XZ, Sun S, Tan CC, Yu JT, Tan L (2017) The Role of ADAM10 in Alzheimer's Disease. J Alzheimers Dis 58:303-322. PMID: 28409746 {Medline}
